# Multiple immunodominant O-epitopes co-expression in live attenuated *Salmonella* serovars induce cross-protective immune responses against *S*. Paratyphi A, *S*. Typhimurium and *S*. Enteritidis

**DOI:** 10.1371/journal.pntd.0010866

**Published:** 2022-10-13

**Authors:** Pei Li, Ke Zhang, Ting Lei, Zuoyong Zhou, Hongyan Luo

**Affiliations:** College of Veterinary Medicine, Southwest University, Chongqing, China; University of Maryland School of Medicine, UNITED STATES

## Abstract

*Salmonella enterica* subsp. *enterica* (*S*. *enterica*) is a significant public health concern and is estimated to cause more than 300,000 deaths annually. Nowadays, the vaccines available for human Salmonellosis prevention are all targeting just one serovar, i.e., *S*. Typhi, leaving a huge potential risk of *Salmonella* disease epidemiology change. In this study, we explored the strategy of multiple immunodominant O-epitopes co-expression in *S*. *enterica* serovars and evaluated their immunogenicity to induce cross-immune responses and cross-protections against *S*. Paratyphi A, *S*. Typhimurium and *S*. Enteritidis. We found that nucleotide sugar precursors CDP-Abe and CDP-Par (or CDP-Tyv) could be utilized by *S*. *enterica* serovars simultaneously, exhibiting O2&O4 (or O4&O9) double immunodominant O-serotypes without obvious growth defects. More importantly, a triple immunodominant O2&O4&O9 O-serotypes could be achieved in *S*. Typhimurium by improving the substrate pool of CDP-Par, glycosyltransferase WbaV and flippase Wzx via a dual-plasmid overexpressing system. Through immunization in a murine model, we found that double or triple O-serotypes live attenuated vaccine candidates could induce significantly higher heterologous serovar-specific antibodies than their wild-type parent strain. Meanwhile, the bacterial agglutination, serum bactericidal assays and protection efficacy experiments had all shown that these elicited serum antibodies are cross-reactive and cross-protective. Our work highlights the potential of developing a new type of live attenuated *Salmonella* vaccines against *S*. Paratyphi A, *S*. Typhimurium and *S*. Enteritidis simultaneously.

## Author summary

Currently, vaccines available for human Salmonellosis prevention are mostly against one serovar, i.e., *S*. Typhi, leaving a significant risk of *Salmonella* disease epidemiology change. Consequently, more efforts are needed to develop vaccine candidates against other medically important but somehow less concerned *S*. *enterica* serovars, such as *S*. Paratyphi A, *S*. Typhimurium and *S*. Enteritidis. Evidence reveals that *S*. Paratyphi A is taking the place of *S*. Typhi as the leading cause of “enteric fever” in South Asia, and *S*. Typhimurium and *S*. Enteritidis are the leading causing of “Invasive Non-typhoidal *Salmonella* disease” in sub-Saharan Africa. One of the solutions is to increase the cross-immunogenicity and cross-protections of potential *S*. *enterica* vaccine candidates. *Salmonella* outer membrane O-antigen polysaccharide is a well-known protective antigen, and its diverse structure is serotype specific. More precisely, *S*. Paratyphi A, *S*. Typhimurium and *S*. Enteritidis are characterized by O2, O4 and O9 O-serotypes. In this study, we aimed to combine the immunodominant O2, O4 and O9 O-epitopes into one *S*. *enterica* serovar and evaluated its cross-immune responses in a murine model. Our results indicated that the rational design of O-antigen structure in live attenuated *Salmonella* vaccines is a promising strategy to induce effective cross-protections against *S*. Paratyphi A, *S*. Typhimurium and *S*. Enteritidis.

## Introduction

*Salmonella* belongs to the family Enterobacteriaceae and is a medically important pathogen for both humans and animals. Based on its surface antigenic composition, *Salmonella* is currently divided into more than 2600 serotypes [1]. However, 99% of human and animal infections are caused solely by one subspecies, *Salmonella enterica* subsp. *enterica* (*S*. *enterica*). *S*. *enterica* is estimated to cause more than 300,000 deaths annually [2,3], mostly in developing countries. According to their clinical manifestations and presentations, *S*. *enterica* has traditionally been divided into ‘typhoidal serovars’ and ‘non-typhoidal serovars’ [4,5]. For example, human host-restricted *S*. *enterica* serovar Typhi (*S*. Typhi) and Paratyphi A (*S*. Paratyphi A) are the leading causes of systemic infections known as typhoid and paratyphoid fever [2], respectively. In contrast, broad host-ranged *S*. *enterica* serovar Typhimurium (*S*. Typhimurium) and Enteritidis (*S*. Enteritidis) generally induce self-limiting gastroenteritis in healthy individuals [6]. However, non-typhoidal serovars may become invasive when the host are infants, young children or immunocompromised adults, causing a life-threatening infection involving the bloodstream, meninges, and other normally sterile sites [7]. Invasive Non-typhoidal *Salmonella* (iNTS) disease is a severe illness with a case fatality ratio of approximately 15%. In sub-Saharan Africa, where the iNTS is observed to be a particular threat [8–10], *S*. Typhimurium and *S*. Enteritidis were the most frequently isolated iNTS pathogens [8], accounting for more than 80%. Unfortunately, widespread antimicrobial resistance among iNTS isolates is threatening the effectiveness of amenable antibiotic treatments [11]. To date, vaccines are regarded as one of the most economical and effective ways to prevent salmonellosis.

Immunity to *Salmonella*, induced by natural infection or vaccination, is serotype-specific [12]. In *S*. *enterica* serovar, this serotype specificity is largely determined by the O-antigen polysaccharide [13] or Vi capsule [14]. The Vi capsule is produced by *S*. Typhi, while the O-antigen is widely found in other *S*. *enterica* serovars. The O-antigen polysaccharide is the outermost part of the lipopolysaccharides (LPS), which is a structurally diverse polymer and repeats in a diverse range of numbers. The LPS is found exclusively in the outer leaflet of the *Salmonella* membrane [15]. Approximately 2×10^6^ LPS molecules cover ∼75% of the cell surface, thus resulting in a formidable barrier limiting the antibodies from accessing the bacterial surface [16]. Nowadays, the only licensed single antigen vaccine against *Salmonella* infections is rationally based on their surface polysaccharides, i.e., the Vi capsule [17] or Vi capsule glycoconjugate vaccines [18]. However, multi-valent strategies are being explored in clinical development. For example, a bivalent outer membrane vesicle approach, also referred to as Generalized Modules for Membrane Antigens (GMMA) [19], targets *S*. Enteritidis and *S*. Typhimurium, and a trivalent glycoconjugate approach targets *S*. Enteritidis, *S*. Typhimurium, and *S*. Typhi [20,21]. Both of them involve in O-antigens formulation. Consequently, an OAg-based vaccine covering the other frequently isolated strains (i.e., *S*. Paratyphi A, *S*. Typhimurium and *S*. Enteritidis) is predictably desirable.

**Fig 1:**
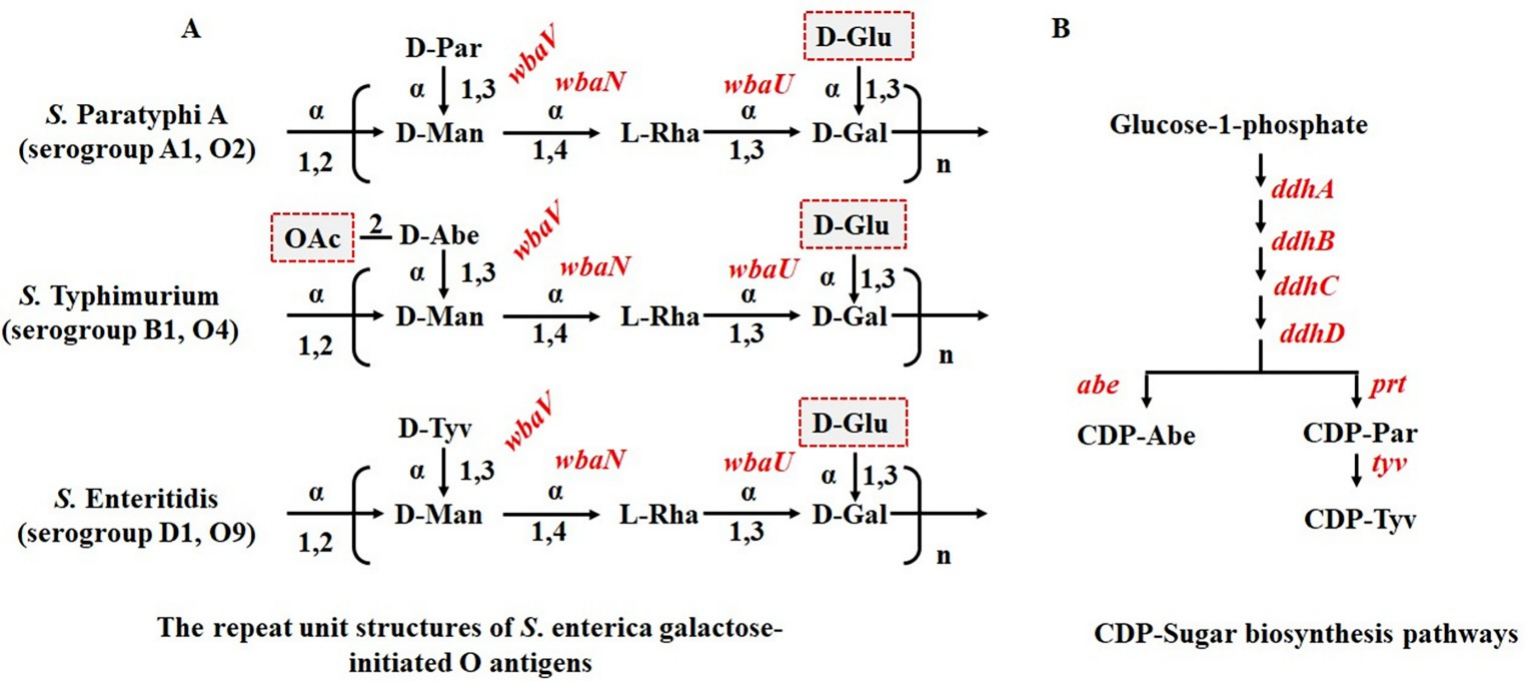
*S. enterica* group A1, B1 and D1 O-unit structures and the CDP-Sugar biosynthesis pathways. (A) Square brackets denoted a single O-unit repeat. The arrows represented the linkages with the linkage types and the glycosyltransferase (GT) indicated. Note that the *S*. Paratyphi A, *S*. Enteritidis and *S*. Typhimurium all share a common trisaccharide backbone. The side-branch sugar of group A1, B1 and D1 was Par, Abe and Tyv, respectively, creating epitopes O2, O4 and O9. Note that the O-antigen galactose can be variably glucosylated, creating epitopes O:12 and O:1. Further, S. Typhimurium can variably O-acetylate its abequose in the C-2 position, creating epitope O:5. (B) Gene responsible for each glycosidic linkage synthesis was indicated adjacent to each arrow. The CDP-Sugars synthesis pathways are all initiated from glucose-1-phosphate. The genes involved in each reaction step of pathways were shown alongside the arrows. Abbreviations: Abe, abequose; Tyv, tyvelose; D-Man, D-mannose; D-Gal, D-galactose; L-Rha, L-rhamnose.

Thanks to the research work of monoclonal antibodies against *S*. *enterica* serogroups A to E, the immunogenic properties of *Salmonella* O-antigen are now clear [22]. The non-specific O-epitopes 1 and 12 are mainly attributed to the O-antigen common trisaccharide backbone 2)-α-Man(1→4)-α-Rha-(1→3)-α-Gal-(1→, which is shared by *S*. Paratyphi A, *S*. Typhimurium and *S*. Enteritidis [23]. However, the immunodominant serovar-specific O-epitopes are largely confined to the paratose (Par), abequose (Abe) and tyvelose (Tyv) side-branch sugars (Fig 1A), namely, O2 (*S*. Paratyphi A, serogroup A1, α-Par(1→3)Man), O4 (*S*. Typhimurium, serogroup B1, α-Abe(1→3)Man) and O9 (*S*. Enteritidis or *S*. Typhi, serogroup D1, α-Tyv(1→3)Man). Passive protection studies demonstrated that IgG or IgM directed against the O2, O4 or O9 O-epitopes played an important role in disease prevention [24]. Consequently, the O-antigenic characteristics of *S*. Paratyphi A, *S*. Typhimurium and *S*. Enteritidis are hereafter referred to as O2, O4 and O9, rather than their full O-antigenic formulae.

The O-antigen gene cluster of *S*. Paratyphi A, *S*. Typhimurium and *S*. Enteritidis are all located between the *galF* and *gnd* gene in the chromosome [25,26] (S1 Fig). The main differences among these gene clusters are the regions responsible for synthesizing the 3,6-dideoxyhexosyl side-branch sugars, and the side-branch sugars are transferred from CDP-sugar precursors by glycosyltransferase WbaV [27,28]. The CDP-sugars synthesis pathways are all initiated from glucose-1-phosphate but diverge after DdhD. CDP-abequose synthase (Abe) and CDP-paratose synthase (Prt) reduce the keto group at C-4 to give either the galactose stereochemistry for CDP-Abe or the glucose stereochemistry for CDP-Par. CDP-Par 2-epimerase (Tyv) is responsible for the synthesis of CDP-tyvelose [27] (Fig 1B). Meanwhile, the Wzx is a multi-transmembrane protein with enormous sequence diversity that flips oligosaccharide substrates with varying degrees of preference [29,30].

Previously, we and others have shown that the immunodominant O-epitope of one *S*. *enterica* strain could be converted to another via chromosomally genetic manipulation [31–33]. For example, the O4 O-serotype in *S*. Typhimurium could be converted into O9 of *S*. Enteritidis by replacing the gene *abe* with *prt*-*tyv*_D1_ without sacrificing its immunogenic properties [31,33]. Similarly, *S*. Typhimurium with *abe-wzx*_*B1*_*-wbaV*_*B1*_ gene replaced by *prt-tyv*_A1_*-wzx*_A1_*-wbaV*_A1_ from *S*. Paratyphi A could convert its original O4 O-serotype into O2 and still retained an excellent immunogenic property [32]. So, we later come up with an interesting question. Could it be possible to simultaneously express immunodominant O2, O4 and O9 O-epitopes in one strain?

In this study, we explored the possibility of exhibiting O2, O4 and O9 immunodominant O-serotypes simultaneously in *S*. *enterica* serovars. The O2 & O4 double O-serotypes phenotype could be easily achieved in either *S*. Paratyphi A or S. Typhimurium background. So did the O4 & O9 double O-serotypes phenotype in *S*. Enteritidis or *S*. Typhimurium background. However, we encountered difficulties in chromosomally co-expressing O2 & O4 & O9 triple O-epitopes, which suggested *S*. *enterica* serovars exhibited different preferences over CDP-Par, CDP-Abe and CDP-Tyv precursors. Unexpectedly, increasing the pool of CDP-Par precursor and the synthesis of WbaV glycosyltransferase and Wzx flippase in *S*. Typhimurium by plasmids overexpression system could result in an O2 & O4 & O9 triple O-serotypes phenotype. A series of live attenuated *S*. *enterica* vaccine candidates were designed and constructed to evaluate their potential cross-immune responses and cross-protections against wild-type *S*. Paratyphi A, *S*. Typhimurium and *S*. Enteritidis. Our research highlights the strategy of manipulating bacteria surface polysaccharide epitopes to generate live attenuated vaccines offering multi-serotype protections.

## Materials and methods

### Ethics statement

All animal experiments were conducted in compliance with the Animal Welfare Act and regulations stated in the Guide for the Care and Use of Laboratory Animals, which was approved by the Institutional Animal Care and Use Committee (IACUC) of Southwest University (IACUC-20210525-02).

### Bacteria, plasmids, and culture conditions

The bacteria and plasmids used in this study are listed in Table 1. *E*. *coli*, *S*. *enterica* serovars and their derivatives were aerobically grown at 37°C in Luria-Bertani (LB) broth or on LB agar. Allelic exchange in *S*. *enterica* serovars was achieved by *sacB* gene-based counter selection on LB agar plates that contained 10% sucrose with no sodium chloride added and incubated at 30°C [34]. When necessary, chloramphenicol was added at 25 μg/ml to select transconjugants. D-Alanine (D-Ala) (50 μg/ml) was added for the growth of Δ*alr* Δ*dadB* strains [35]. Diaminopimelic acid (DAP) was added at 50 μg/ml for the growth of Δ*asd* mutant χ7213 [36]. *In vitro* growth rates of *Salmonella* strains were determined by optical density measurements.

**Table 1:** Bacterial strains and plasmids used in this study.

Strains or Plasmids	Description*	Source
*Salmonella* and *E*.*coli*		
S356	*S*. Paratyphi A, O2	[29]
S100	*S*. Typhimurium, O4	[30]
S246	*S*. Enteritidis, O9	[30]
L001	SA-*ddhc*::*abe*, O2&O4, derived from S356	This study
L002	ST-*ddhc*::*prt*_A1_, O2&O4, derived from S100	This study
L003	SE-*ddhc*::*abe*, O4&O9, derived from S246	This study
L004	ST-*ddhc*::*prt*_*D1*_*-tyv*_D1_, O4&O9, derived from S100	This study
K008	ST-Δ*alr* Δ*dadB* Δ*recF* Δ*asd*, O4, derived from S100	Lab stored
L056	ST-Δ*alr* Δ*dadB* Δ*recF* Δ*asd* (pSC101-*asd*-O2), O2&O4	This study
L057	ST-Δ*alr* Δ*dadB* Δ*recF* Δ*asd* (p15a-*dadB*-O9), O4&O9	This study
L058	ST-Δ*alr* Δ*dadB* Δ*recF* Δ*asd* (pSC101-*asd*-O2, p15a-*dadB*-O9), O2&O4&O9	This study
S738	ST-Δ*cya* Δ*crp*, O4, derived from S100	[28]
L015	SE-Δ*cya* Δ*crp*, O9, derived from S246	[28]
L008	ST-ddhc::*prt*_A1_ Δ*cya* Δ*crp*, O2&O4, derived from L001	This study
L009	SE-*ddhc*::*abe* Δ*cya* Δ*crp*, O4&O9, derived from L004	This study
K013	ST-Δ*alr* Δ*dadB* Δ*recF* Δ*cya* Δ*crp* Δ*asd*, O4, derived from S100	Lab stored
L083	ST-Δ*alr* Δ*dadB* Δ*recF* Δ*cya* Δ*crp* Δ*asd* (pSC101-*asd*-O2, p15a-*dadB*-O9), O2&O4&O9, derived from K013	This study
χ7232	*E*. *coli endA1 hsdR17* (r_K_-, m_K_*+*) *glnV44 thi-1 recA1 gyrA relA1 Δ*(*lacZYA-argF*)*U169 λpir deoR* (ϕ*80dlac* Δ(*lacZ*)*M15*)	[28]
χ7213	*E*. *coli thi-1 thr-1 leuB6 glnV44 fhuA21 lacY1 recA1 RP4-2-Tc*::Mu λ*pir* Δ*asdA4* Δ*zhf-2*::Tn*10*	[28]
Suicide plasmids		
pRE112	*sacB* mobRP4 R6K *ori* Cm+	[28]
pSW005	pSC101 *ori*, *asd*, P_trc_ promoter, 5ST1T2 terminator (pSC101-*asd*)	Lab stored
pSW049	p15a *ori*, *dadB*, P_trc_ promoter, 5ST1T2 terminator (p15a-*dadB*)	Lab stored
pSW084	*prt*_A1_*-tyv*_A1-_*wzx*_A1-_*wbaV*_A1_ expression, derived from pSW005, named pSC101-*asd*-O2	This study
pSW096	*prt*_*D1*_*-tyv*_D1-_*wzx*_D1-_*wbaV*_D1_ expression, derived from pSW048, named p15a-*dadB*-O9	This study
pHY005	*ddhc*::*prt*_A1_ insertion mutation construction, derived from pRE112	This study
pHY006	*ddhc*:: *abe* insertion mutation construction, derived from pRE112	This study
pHY007	*ddhc*::*prt-tyv*_D1_ insertion mutation construction, derived from pRE112	This study

* The O-antigen serotype information for each applicable strain only showed its immunodominant O-serotype.

### Molecular and genetic procedures

Molecular biology techniques were performed following standard methods [37], and details regarding the primers used in this study are listed in the S1 Table. DNA concentration and purity were measured using a Nanodrop ND-2000 spectrophotometer (Thermo Fisher Scientific), and DNA fragments were cyclized by Circular Polymerase Extension Cloning (CPEC) method [38].

Insertion or deletion mutant strains construction. As antibiotic resistance is not permitted in live vaccine strains, *sacB* gene-based sucrose counter-selectable suicide vectors were used to construct unmarked insertion or deletion mutations in *S*. *enterica* serovars [34]. Take the construction of L001 (SA-*ddhc*::*abe*) as an example; the upstream and downstream homologous regions were amplified from *S*. Paratyphi A using primer pairs P11/P12 and P15/P16. The *abe* gene was amplified from *S*. Typhimurium using primer pairs P13/P14 and the suicide vector backbone was amplified from pRE112 using primer pairs P1/P2. After purification, these three fragments were cyclized by CPEC method, resulting in a new suicide plasmid pHY005 (112-*ddhc*::*prt*_A1_). The conjugational transfer of pHY005 to *S*. Paratyphi A was performed using the suicide vector donor strain χ7213 [39]. Transconjugants with the first homologous recombination event were selected on chloramphenicol agar without supplemental DAP. The positive clones were inoculated in fresh LB media without chloramphenicol addition. The second homologous recombination event, resulting in excision of the suicide vector from the *S*. Paratyphi A chromosome, was selected on 10% sucrose LB plates without sodium chloride and grown at 30°C. Successful gene insertion mutations were confirmed by PCR screening and DNA sequencing. Other insertion mutations are constructed following the same procedures. The insertion mutations constructed for this study are illustrated in S2 Fig, and the primer pairs used for each DNA fragment amplification were labeled accordingly. As for the *crp* and *cya* gene deletion mutations, the suicide plasmids 112-Dcrp and 112-Dcya were constructed previously [33]. The subsequent deletion mutation processes were similar to those described above for the insertion mutations. All successful gene deletion or insertion mutations were confirmed by DNA sequencing.

Recombinant plasmids construction. Briefly, *prt*, *tyv*, *wzx* and *wbaV* genes were cloned from *S*. Paratyphi A or *S*. Enteritidis using the same primer pairs P19/P20. To distinguish them from each other, we added an "A1" suffix subscript after each gene cloned from *S*. Paratyphi A, as *S*. Paratyphi A belongs to *S*. *enterica* serogroup A1. Similarly, a “D1” suffix subscript was added to each gene cloned from *S*. Enteritidis, as *S*. Enteritidis belongs to *S*. *enterica* serogroup D1. Next, the vector backbone was cloned from pSC101-*asd* and p15a-*dadB* using the same primer pairs P17/P18, as these two plasmids all contained a Ptrc promoter-multiple cloning site (MCS)-5ST1T2 terminator cassette. After purification, the DNA fragment *prt*_A1_-*tyv*_A1_-*wzx*_A1_-*wbaV*_A1_ and the linearized pSC101-*asd* vector were cyclized by CPEC method, resulting in pSC101-*asd-*O2. Similarly, the DNA fragment *prt*_D1_-*tyv*_D1_-*wzx*_D1_-*wbaV*_D1_ and the linearized p15a-dadB vector were cyclized by CPEC method, resulting in p15a-*dadB-*O9.

### Phenotype characterization of mutant strains

Mutant strains phenotype evaluations included the LPS silver stain and western blot, slide agglutination test, P22 transduction studies, growth rates, motility test, and minimum inhibitory concentration (MIC) test of deoxycholate (DOC) and polymyxin B. These methods were all reported previously, and a complete description of all methods employed for these phenotype evaluations is provided in S1 Text.

### Virulence determination and colonization in mice

Six-week-old female BALB/c mice were purchased from Dashuo Biotechnology Co., Ltd. (Chengdu, China). To determine the 50% lethality dose (LD_50_), bacteria were grown to OD_600_ of 0.8 to 0.9 and harvested by centrifugation at 3,452 × g at room temperature. The centrifuged *S*. Typhimurium, *S*. Enteritidis or their derivatives were resuspended and adjusted to the appropriate OD_600_ value by buffered saline with gelatin (BSG) [40]. In contrast, the centrifuged *S*. Paratyphi A or its derivatives were resuspended and adjusted to the appropriate OD_600_ value by 10% hog gastric mucin (Sigma). Six mice per group were infected orally with 20 μl of BSG or intraperitoneally injected with 500 μl 10% hog gastric mucin containing various doses of bacteria, ranging from 1 × 10^4^ CFU to 1 × 10^8^ CFU. Mice were monitored for mortality or signs of significant morbidity daily. The LD_50_ was calculated using the method of Reed and Muench. To evaluate colonization, three mice per group were orally inoculated with 20 μl of BSG containing 1 × 10^9^ CFU bacteria. On days 4 and 8 post-inoculation, Peyer’s patches, spleen and liver samples were collected. Samples were homogenized, dilutions were plated onto MacConkey and LB agar to determine viable counts.

### Vaccination and immune response measurement

Thirty mice per group were vaccinated orally on day 0 with 20 μl BSG containing approximately 1 × 10^9^ CFU vaccine strains and boosted on day 14 with the same dose. Blood samples and vaginal secretions were collected from randomly selected twelve mice in each group on day 28 after the booster immunization. On day 35, mice in each group were randomly subdivided into three small subgroups; mice in each subgroup were challenged orally with 5 × 10^7^ CFU of *S*. Typhimurium and *S*. Enteritidis or injected intraperitoneally with 1 × 10^4^ CFU of *S*. Paratyphi A (~100 times the LD_50_) [40,41]. *S*. Paratyphi A, *S*. Typhimurium and *S*. Enteritidis LPS were purified as described previously [42] and used as coating antigens to measure the immune responses. IgM, IgG and IgA antibodies specific to *S*. Paratyphi A, *S*. Typhimurium and *S*. Enteritidis LPS in the serum or vaginal secretions were measured using the quantitative enzyme-linked immunosorbent assay (ELISA) as described previously [43]. Antibody concentrations were calculated based on absorbance values and the standard curve.

### Serum bactericidal activity assay

The serum bactericidal assay (SBA) was performed as previously described with a few modifications [44]. Briefly, log-phase cultures of *S*. Paratyphi A, *S*. Typhimurium and *S*. Enteritidis were grown to an OD_600_ of 0.6 in LB broth. After centrifugation and resuspension, the log-phase cultures were diluted in PBS to a concentration of approximately 1× 10^4^ CFU/ml. Sera samples from vaccinated mice were serially diluted from 1:100 to 128,000, and nonimmune sera were serially diluted from 1: 10 to 1,280. Optimal SBA results were achieved by combining 25 μl of active baby rabbit complement (BRC) (25% final concentration) with 15 μl of PBS, 50 μl of diluted mice pooled sera, and 10 μl of diluted bacteria (~350 CFU). In total, 10 μl of the mixture from each well was spread on LB agar plates after 60 min to assess the bactericidal activity. The spread LB agar plates were incubated overnight at 37°C, and the viable CFU were counted the next day. The negative control contained only bacteria and complement. The serum bactericidal antibody titer was defined as the reciprocal of the highest serum dilution that produced >50% killing in relation to the killing observed for the control wells. The titers were determined from the mean bacterial count from triplicate wells.

### Statistical analysis

Data were analyzed using GraphPad Prism 5 software (Graph Software, San Diego, CA) by one-way or two-way ANOVA of variance followed by Tukey’s multiple-comparison post-test. Kaplan-Meier survival curve comparisons were calculated by comparing two groups at each time point through the log-rank (Mantel-Cox) test. The data were expressed as the means ± SD. *P*<0.05 was considered statistically significant.

## Results

### *S*. *enterica* mutants exhibiting double or triple immunodominant O-serotypes

**Fig 2:**
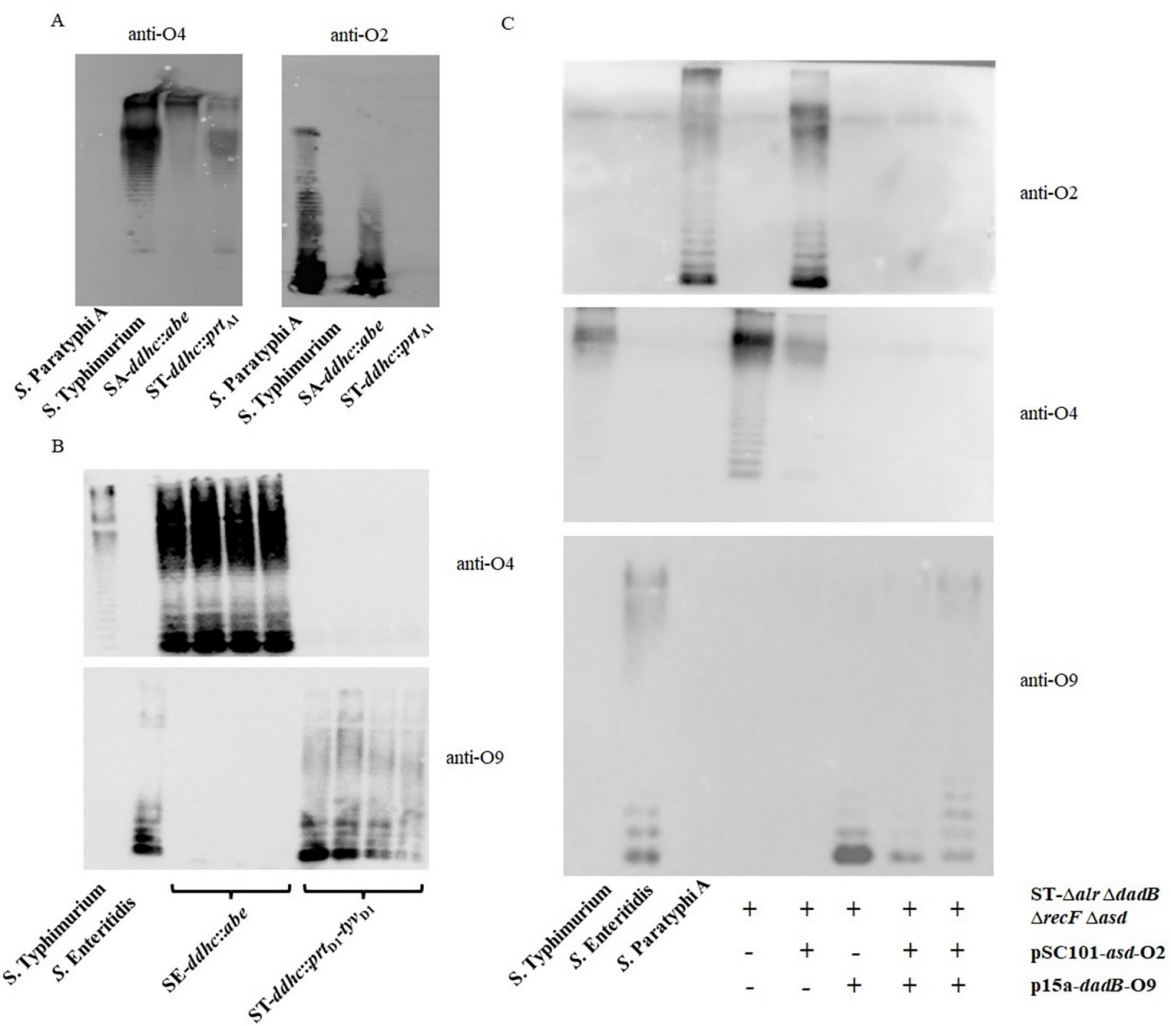
Immunoblots analysis. LPS was extracted from the *S*. *enterica* mutants exhibiting O2&O4 (A), O4&O9 (B) and O2&O4&O9 (C) O-serotypes and separated on a 12.5% (w/v) polyacrylamide gel by tricine-SDS-PAGE. Samples were then transferred to a nitrocellulose membrane for immunoblotting with anti-Par O2, anti-Abe O4 or anti-Tyv O9 antisera. The detected bands corresponded to LPS with different side-branch O-unit repeats.

The primary goal of this study is to try to exhibit O2, O4 and O9 O-serotypes simultaneously in one *S*. *enterica* serovar. To begin with, we explore the possibility of co-expressing O2 & O4 or O4 & O9 O-epitopes first. According to the O-antigen gene cluster analysis (S1 Fig), the major difference among *S*. Paratyphi A, *S*. Typhimurium and *S*. Enteritidis lie in the genes responsible for the synthesis of the side-branch sugar (Fig 1B), i.e., the *abe*, *prt* and *tyv* genes for CD-Abe, CD-Par and CDP-Tyv biosynthesis respectively. Consequently, we inserted the *abe* gene from *S*. Typhimurium between the *ddhc* and *prt* gene of *S*. Paratyphi A to test the potential of co-expressing O2 and O4 O-epitopes (S2A Fig). The results of bacterial agglutination (Table 2) and western blot (Fig 2A) showed that the SA-*ddhc*::*abe* mutant could react positively to O2 and O4 antisera, respectively, indicating the co-existence of O2 and O4 O-epitopes in the outer membrane of SA-*ddhc*::*abe* mutant. To be more persuasive, we inserted the *prt*_A1_ gene from *S*. Paratyphi A similarly between the *ddhc* and *abe* gene of *S*. Typhimurium (S2B Fig) to see if these co-expressing phenomena still existed. Consistently, the bacterial agglutination (Table 2) and western blot results (Fig 2A) all indicated that the ST-*ddhc*::*prt*_A1_ mutant exhibited O2 and O4 O-epitopes simultaneously. At the same time, the *abe* gene was inserted between the *ddhc* and *prt*_D1_ gene of *S*. Enteritidis (S2C Fig) and the *prt*_D1_-*tvy*_D1_ gene was inserted between the *ddhc* and *abe* gene of *S*. Typhimurium (S2D Fig). Unsurprisingly, either SE-*ddhc*::*abe or* ST-*ddhc*::*prt*_*D1*_*-tyv*_*D1*_ could independently agglutinate with O4 and O9 antisera obviously (Table 2). However, we did not observe clear anti-O9 positive bands of SE-*ddhc*::*abe* or clear anti-O4 positive bands of ST-*ddhc*::*prt*_*D1*_*-tyv*_*D1*_ in western blot results (Fig 2B). Unfortunately, we failed to obtain a *S*. Paratyphi A, *S*. Typhimurium or *S*. Enteritidis mutant that could express O2, O4 and O9 O-epitopes simultaneously through chromosomally genetic modifications.

We have noticed that *S*. Enteritidis possess both functional *prt* and *tyv* genes and it turns out to be a dominant O9 O-serotype, while the *tyv* gene in *S*. Paratyphi A is frameshift and yet it turns out be a dominant O2 O-serotype. So, we speculate that when *prt* and *tyv* are co-expressed at a similar level, the CDP-Par will be mostly converted into CDP-Tyv, resulting in an O9 dominant O-serotype. Therefore, we hypothesize that it might be possible to express O2, O4 and O9 O-epitopes simultaneously by improving the CDP-Par substrate pool. Based on that assumption, we have built a dual-plasmid expression system. The ST-Δ*alr* Δ*dadB* Δ*recF* Δ*asd* mutant was constructed deliberately to harbor pSC101-*asd* and p15a-*dadB* plasmids without antibiotic selection pressure [35]. The *prt*_A1_*-tyv*_A1_*-wbaV*_A1_*-wzx*_A1_ genes from *S*. Paratyphi A were cloned into pSC101-*asd*, resulting in pSC101-*asd-*O2, and the *prt*_D1_*-tyv*_D1_*-wbaV*_D1_*-wzx*_D1_ genes from *S*. Enteritidis were cloned into p15a-*dadB*, resulting in p15a-*dadB-*O9. As *tyv*_A1_ possesses a loss-of-function mutation, when pSC101-*asd-*O2 and p15a-*dadB-*O9 were transferred into ST-Δ*alr* Δ*dadB* Δ*recF* Δ*asd*, the *prt* gene were comparatively overexpressed. Except for anti-O9, we did not observe anti-O2 or anti-O4 positive bands of ST-Δ*alr* Δ*dadB* Δ*recF* Δ*asd* (pSC101-*asd*-O2, p15a-*dadB-*O9) in western blot results (Fig 2C). However, the bacterial agglutination assays had clearly shown that, in addition to O4 antiserum, ST-Δ*alr* Δ*dadB* Δ*recF* Δ*asd* harboring pSC101-*asd*-O2 or p15a-*dadB-*O9 could agglutinate with O2 or O9 antiserum, respectively (Table 2). Most importantly, ST-Δ*alr* Δ*dadB* Δ*recF* Δ*asd* (pSC101-*asd*-O2, p15a-*dadB-*O9) could agglutinate with O2, O4 and O9 antisera independently, which indicated that we had successfully exhibited O2, O4 and O9 O-serotypes simultaneously in *S*. Typhimurium outer membrane.

**Table 2:** Bacterial agglutination assays.

Serum^a^ Bacteria	O2	O4	O9	Serotype
S356 *S*. Paratyphi A	**+++** ^**b**^	**-**	**-**	O2
S100 *S*. Typhimurium	**-**	**+++**	**-**	O4
S246 *S*. Enteritidis	**-**	**-**	**+++**	O9
L001 SA-*ddhc*::*abe*	**++**	**++**	**-**	O2&O4
L002 ST-*ddhc*::*prt*_A1_	**++**	**+**	**-**	O2&O4
L003 SE-*ddhc*::*abe*	**-**	**++**	**++**	O4&O9
L004 ST-*ddhc*::*prt-tyv*_D1_	**_**	**+**	**+++**	O4&O9
L056 ST-Δ*alr* Δ*dadB* Δ*recF* Δ*asd* (pSC101-*asd*-O2)	**++**	**+**	**-**	O4&O2
L057 ST-Δ*alr* Δ*dadB* Δ*recF* Δ*asd* (p15a-*dadB*-O9)	**-**	**++**	**++**	O4&O9
L058 ST-Δ*alr* Δ*dadB* Δ*recF* Δ*asd* (pSC101-*asd*-O2, p15a-*dadB*-O9)	**++**	**+**	**++**	O2&O4&O9

^a^ serum used in this assay were anti-Par O2, anti-Abe O4 and anti-Tyv O9 antiserum.

^b^ agglutination observed immediately was indicated as “+++”, within 30 sec as “++”, more than 1 min as “+”.

### Phenotype characterizations of *S*. *enterica* mutants

**Fig 3:**
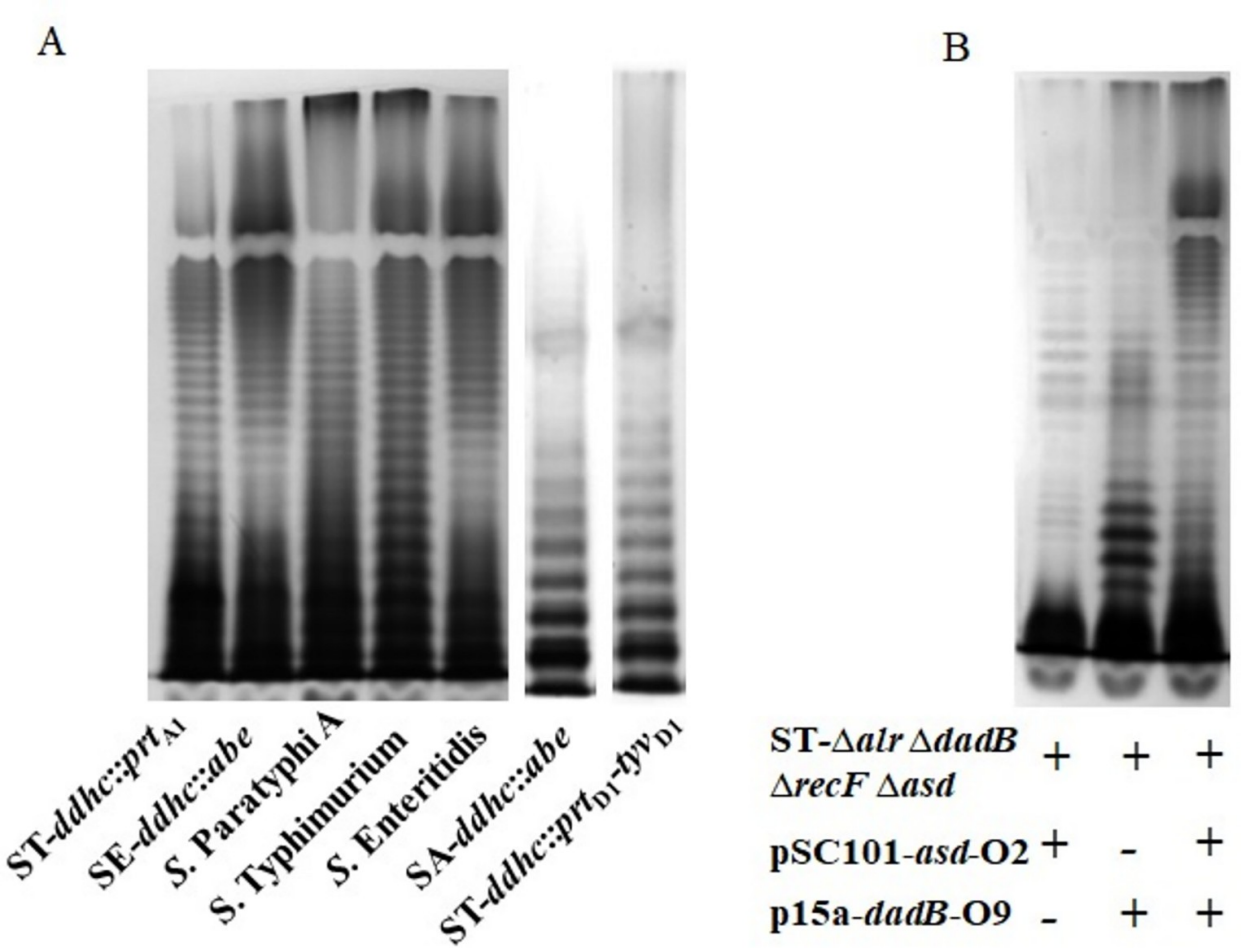
LPS profiles of *S*. *enterica* mutants. *S*. enterica mutants exhibiting double or triple immunodominant O-serotypes were constructed by chromosomally gene insertion mutations (A) or plasmid overexpressing systems (B). The LPS extracted from these mutant strains was separated on a 12.5% (w/v) polyacrylamide gel by tricine-SDS-PAGE and visualized by the silver staining. Bands represented the LPS with different numbers of O-unit repeats.

**Fig 4:**
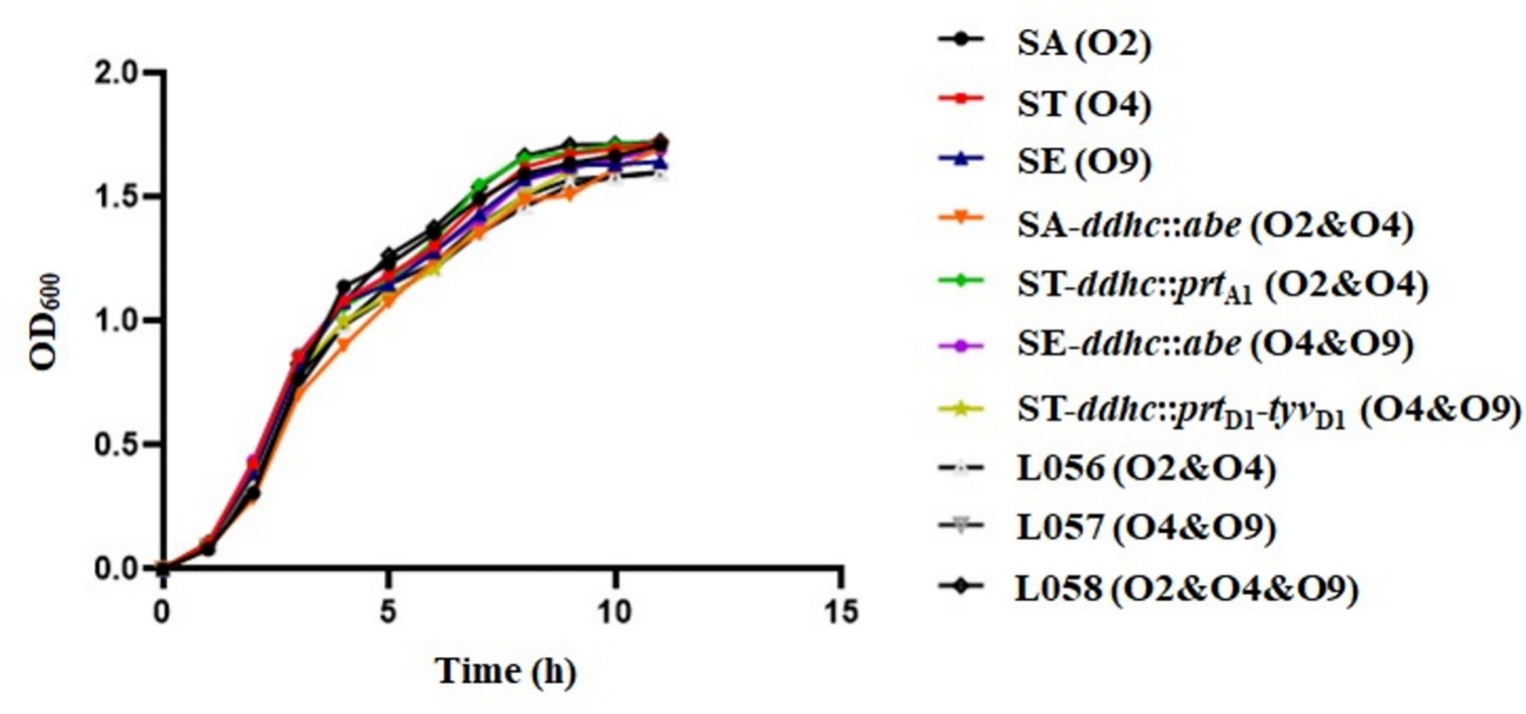
Growth curves. In vitro growth rates of *S*. enterica and its derivatives were measured by optical density measurements as an OD_600_ value at multiple time points.

As we have achieved double or triple immunodominant O-epitopes co-expression in *S*. *enterica* mutants, we would like to know whether or not these O-serotypes’ co-existence would influence their phenotype characterizations. Firstly, the LPS profiles of these mutant strains were visualized by silver staining. Most mutants can synthesize a full length of LPS when compared with their wild-type parent strains, except for ST-Δ*alr* Δ*dadB* Δ*recF* Δ*asd* (pSC101-*asd*-O2, p15a-*dadB*), which exhibited a decrease in LPS synthesis (Fig 3). Next, the accessibilities of mutants’ O-antigen structure for Phage P22 were examined. The number of transductions obtained from double or triple O-serotypes exhibiting mutants was similar to that of their wild-type parent strains (Table 3), indicating that their O-antigen backbones were unaffected. Secondly, the growth rate of these mutant strains and their wild-type parent strains were all evaluated, and there was no significant difference between them (Fig 4).

**Table 3:** Transduction efficiencies, MIC of DOC and Polymyxin B, swimming motility and virulence of wild type *Salmonella* and its derivatives.

Strain	Serotype changed^a^	Number of P22 transductants^b^	MIC		Swimming motility (mm)^d^	LD_50_(CFU)
			DOC (mg/ml)^c^	Polymyxin B (μg/ml)		
L001	O2&O4	257 ± 34	20	0.575	14.01 ± 2.254	5.17 x 10^6^
L002	O2&O4	287 ± 31	20	0.575	22.02 ± 4.852	1.10 x 10^6^
L003	O4&O9	384 ± 36	20	0.575	27.33 ± 2.517	1.83 x 10^6^
L004	O4&O9	359 ± 29	20	0.575	25.28 ± 3.605	4.88x 10^6^
L056	O2&O4	243 ± 36	20	0.575	12.21 ± 2.441	5.18 x 10^7^
L057	O4&O9	277 ± 27	20	0.575	18.04 ± 1.908	1.83 x 10^7^
L058	O2&O4&O9	221 ± 18	20	0.575	11.16 ± 1.057	5.38 x 10^7^
S356	O2	314 ± 45	20	1.15	15.71 ± 0.577	1.20^e^ x 10^2^
S100	O4	434 ± 48	20	1.15	29.34± 0.874	1.59 x 10^5^
S246	O9	474 ± 57	20	1.15	30.67 ± 1.517	1.12 x 10^5^

^a^ Immunodominant O-serotype

^b^ The results reflect the numbers of chloramphenicol-resistant colonies obtained after transduction (means ± SD).

^c^ DOC, deoxycholate.

^d^ The average diameter in millimeters (means ± SD).

^e^ The LD_50_ value of wild-type *S*. Paratyphi A is determined in a lethal murine model that requires suspending the bacteria in 5–10% hog gastric mucin and then injecting the suspension intraperitoneally

Meanwhile, their sensitivity to the bile salt DOC and the cationic antimicrobial peptide polymyxin B were tested. The DOC MICs had no difference among these strains, whereas the polymyxin B MICs for double or triple O-epitopes co-expressing mutant strains were twofold lower than their wild-type parent strains. All mutants retained similar swimming motility as their wild type parent strains (Table 3). Taken together, all these results indicated that double or triple O-epitopes co-expressing had a minor influence on the phenotype changes of our constructed *S*. *enterica* mutants.

### Virulence and colonization of the *S*. *enterica* mutants in BALB/c mice

The LD_50_ values of wild-type *S*. Typhimurium S100 and *S*. Enteritidis S246 were approximately 10^5^ CFU, whereas the LD_50_ values of double or triple O-epitopes co-expressing mutant strains were approximately 10^6^ or 10^7^ CFU, showing around 10 times or 100 times attenuation (Table 3). Since *S*. Paratyphi A is a human host-restricted pathogen, its virulence attribute was evaluated in a lethal murine model that requires suspending the bacteria in 5–10% hog gastric mucin and then injecting the suspension intraperitoneally [45]. In this model, the LD_50_ value of wild-type *S*. Paratyphi A was approximately 100 CFU, showing a highly virulent attribute.

**Fig 5:**
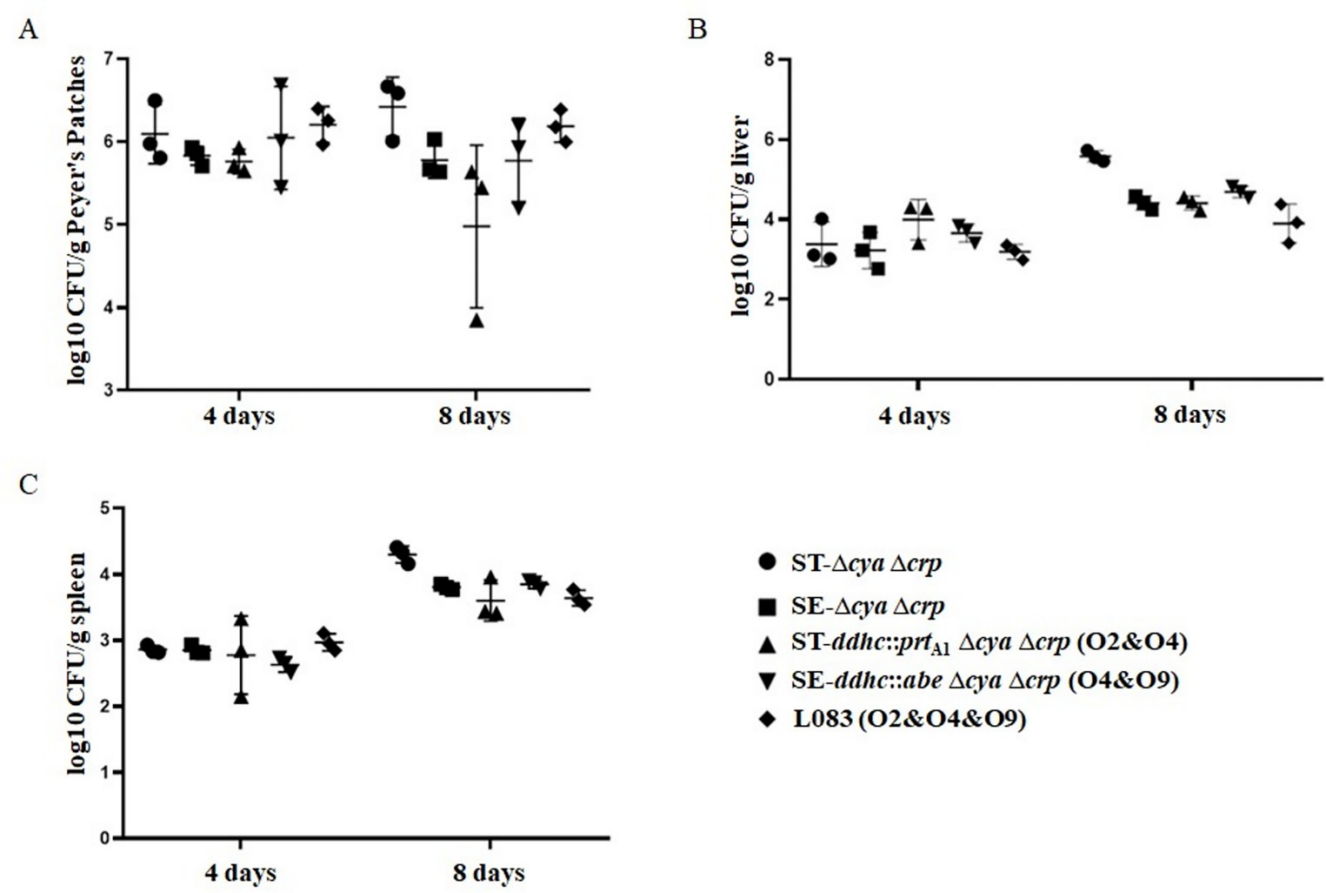
Colonization of murine Peyer’s patches, livers and spleens by live attenuated *S*. enterica vaccines. Colonization of mouse Peyer’s patches (A), livers (B) and spleens (C) 4 and 8 days post-inoculation were shown. The horizontal lines represent the means, and the error bars represent the standard deviation of the means.

Considering the phenotype characterizations and broad host adaption abilities, we select ST-*ddhc*::*prt*_A1_, SE-*ddhc*::*abe* and ST-Δ*alr* Δ*dadB* Δ*recF* Δ*asd* (pSC101-*asd*-O2, p15a-*dadB-*O9) for further potential live attenuated vaccine candidates development. To guarantee their safety in the murine model, we further deleted the *cya* and *crp* global regulators of these mutant strains [46], resulting in L008 (ST-*ddhc*::*prt*_A1_ Δ*cya* Δ*crp*), L009 (SE-*ddhc*::*abe* Δ*cya* Δ*crp*) and L083 [ST-Δ*alr* Δ*dadB* Δ*recF* Δcya Δcrp Δ*asd* (pSC101-*asd*-O2, p15a-*dadB-*O9)], respectively. Again, their O-serotype phenotypes have been reconfirmed, and the deletion mutations of *cya* and *crp* genes did not affect the O-epitopes expression (S3 Fig) but could attenuate these candidates more than 1000 times (LD_50_ > 5 ×10^8^ CFU). To be more briefly, these mutant strains will be hereafter referred to as L008 (O2&O4), L009 (O4&O9) and L083 (O2&O4&O9). The colonization of L008 (O2&O4), L009 (O4&O9) and L083 (O2&O4&O9) in murine Peyer’s patches, spleens, and livers was determined on days 4 and 8 after oral inoculation. All candidates displayed good colonization in Peyer’s patches, livers, and spleens. No deaths occurred during this period (Fig 5).

### Immune responses induced by live attenuated *S*. *enterica* vaccines

To assess the immunogenicity of these vaccine candidates, mice were inoculated orally with approximately 10^9^ CFU of each strain on day 0 and boosted on day 14 with the same doses. Anti-*S*. Paratyphi A, anti-*S*. Enteritidis and anti-*S*. Typhimurium LPS serum antibodies were measured on day 28.

**Fig 6:**
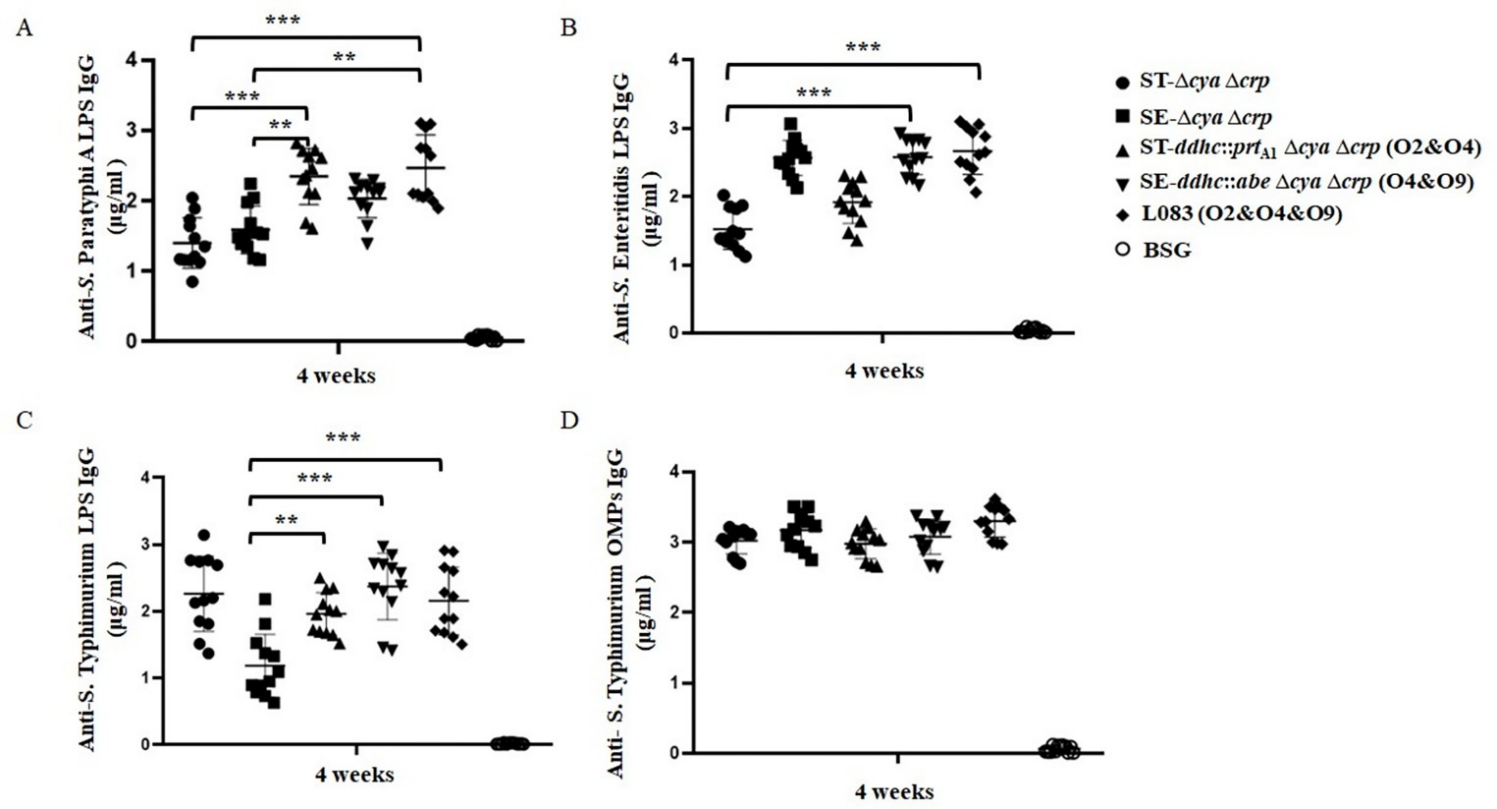
Serum IgG antibody responses. (A) Anti-*S*. Paratyphi A LPS serum IgG levels. Responses that differed from the results in the ST-Δ*cya* Δ*crp* group are noted by asterisks (**, P<0.01; ***, P<0.001). (B) The anti-*S*. Enteritidis LPS serum IgG levels. Responses that differed from the results in the ST-Δ*cya* Δ*crp* group are noted by asterisks (***, P<0.001). (C) Anti-*S*. Typhimurium LPS serum IgG levels. Responses that differed from the results in the SE-Δ*cya* Δ*crp* group are noted by asterisks (**, P<0.01; ***, P<0.001). Antibody concentrations were calculated using a standard curve and all the measured sample concentrations were within the standard curve range. The error bars represent the standard deviation of the means calculated by GraphPad Prism software. These data are representative of at least two independent experiments.

The IgG immune response are depicted in Fig 6. Mice vaccinated with L008 (O2&O4) and L083 (O2&O4&O9) mounted a significantly higher anti-*S*. Paratyphi A LPS immune responses than those of ST-Δ*crp* Δ*cya* or SE-Δ*crp* Δ*cya*. Similarly, mice vaccinated with SE-Δ*crp* Δ*cya*, L009 (O4&O9) and L083 (O2&O4&O9) mounted a significantly higher anti-*S*. Enteritidis LPS immune responses than those of ST-Δ*crp* Δ*cya*. Except for SE-Δ*crp* Δ*cya*, mice vaccinated with other vaccine candidates could all mount a significantly higher anti-*S*. Typhimurium LPS responses. We did observe some cross-immunogenicity between ST-Δ*crp* Δ*cya* and SE-Δ*crp* Δ*cya*, but it had long been proved to be the common trisaccharide backbone and the conserved core oligosaccharide. So far, we did not obtain valid evidence showing that there were detectable cross-immune responses among immunodominant O2, O4 and O9 O-epitopes unless they were co-expressed simultaneously. L008 (O2&O4) could mount a significantly higher anti-*S*. Paratyphi A LPS immune response than those vaccinated with ST-Δ*crp* Δ*cya* (O4), showing an improved cross-immune response against *S*. Paratyphi A. Consistently, L009 (O4&O9) could mount a significantly higher anti-*S*. Typhimurium LPS immune response than those vaccinated with SE-Δ*crp* Δ*cya* (O9), showing an improved cross-immune response against *S*. Typhimurium. Most importantly, L083 (O2&O4&O9) could simultaneously mount a significantly higher anti-*S*. Paratyphi A and anti-*S*. Enteritidis LPS immune response than those vaccinated with ST-Δ*crp* Δ*cya* (O4), showing a good sign of eliciting cross-protections against *S*. Paratyphi A and *S*. Enteritidis. Meanwhile, all vaccine candidates induced a significantly higher IgG2a response than IgG1 (S4 Fig), indicating a predominantly Th1-type response.

We also evaluated the serum IgM (S5 Fig) and vaginal washes IgA antibodies immune responses (S6 Fig). A similar trend of IgM and IgA immune responses has been observed compared to IgG antibodies. Unsurprisingly, L008 (O2&O4), L009 (O4&O9) and L083 (O2&O4&O9) could induce a significantly higher amount of cross-reactive IgM antibodies than their parent strains. However, the elicited IgM antibodies were even higher than their IgG counterpart, which is unexpected. A significantly higher level of secreted IgA antibodies against *S*. Paratyphi A and *S*. Enteritidis were observed in mice vaccinated by L083 (O2&O4&O9) when compared to its parent strain ST-Δ*crp* Δ*cya*. However, L009 (O4&O9) could elicit a higher level of anti-*S*. Enteritidis LPS IgA compared to L083 (O2&O4&O9) and the level of anti-*S*. Paratyphi A LPS IgA was similar between L008 (O2&O4) and L083 (O2&O4&O9). Negative control groups did not mount a detectable immune response. All these ELISA results had shown that our vaccine candidates could induce promising cross-immune responses.

### Antibody-dependent complement-mediated *S*. *enterica* killing

**Fig 7:**
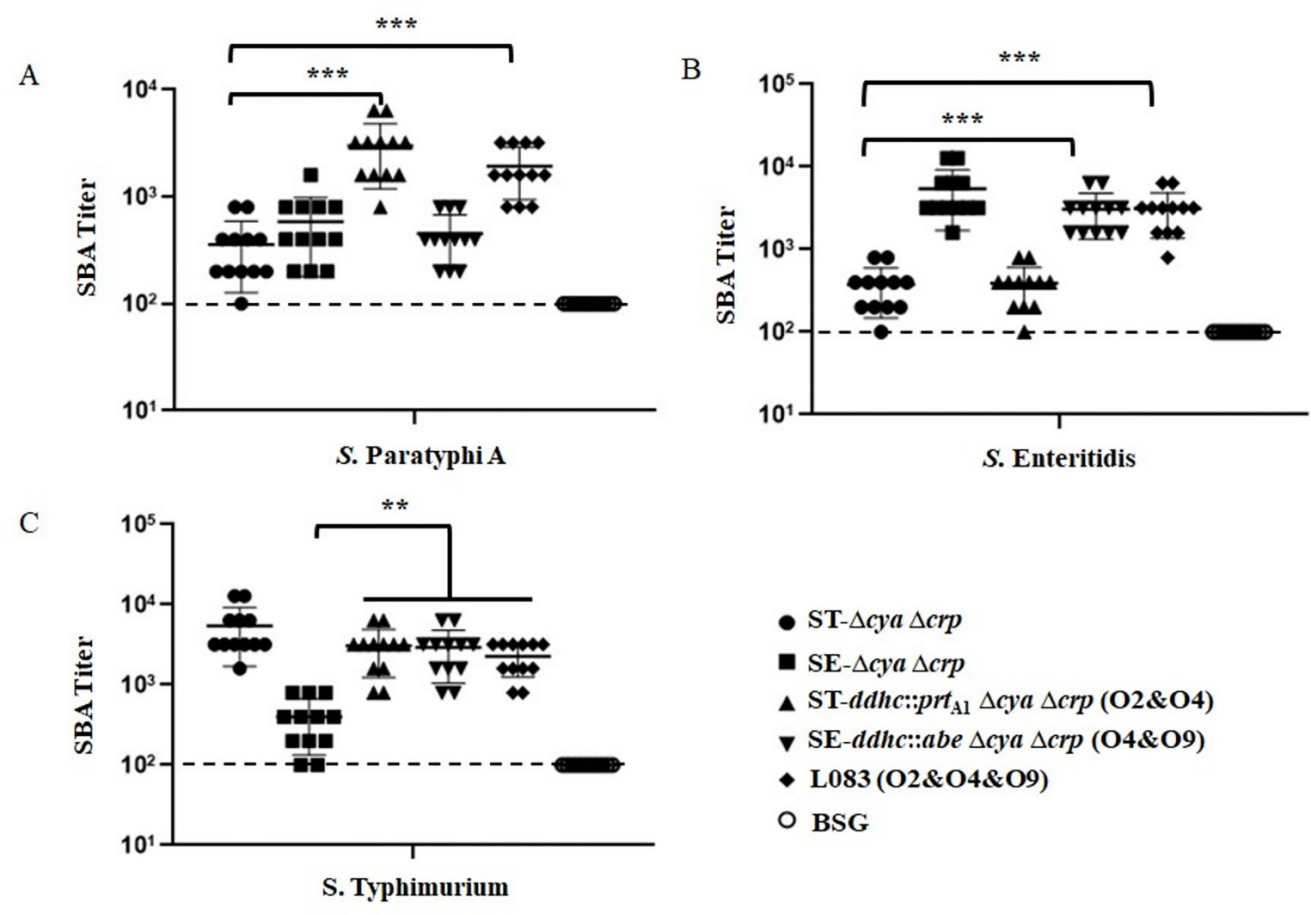
Serum bactericidal activity. Serum bactericidal assays (SBA) were performed with vaccinated mouse serum from the indicated groups against wild-type *S*. Paratyphi A (A), *S*. Enteritidis (B) and *S*. Typhimurium (C). Strains were grown in LB to log phase (OD_600_, 0.4). The error bars represent the standard deviation of the mean titers calculated by GraphPad Prism software. The dashed lines indicate the detection limit of the assay. **, P<0.01; ***, P<0.001.

To evaluate the functional capacities of antibodies induced by our vaccine candidates, we performed the serum bactericidal assays (SBAs) using pooled serum from immunized mice or BSG control. Baby rabbit complement titration with serum samples from immunized mice had a significantly higher complement-mediated killing activity versus those from nonimmunized mice (Fig 7). The serum antibodies induced by ST-Δ*crp* Δ*cya* (O4) or SE-Δ*crp* Δ*cya* (O9) exhibited a high level of SBA activity against its homologous serotype strains. However, they had a limited SBA activity against heterologous serotype strains. On the contrary, the serum antibodies induced by L008 (O2&O4) and L009 (O4&O9) significantly improved SBA activity against heterologous serotype strains *S*. Paratyphi A and *S*. Typhimurium, respectively. More importantly, the bactericidal abilities induced by L083 (O2&O4&O9) covered all tested heterologous serotype strains, significantly improving SBA activity against *S*. Paratyphi A, *S*. Typhimurium and *S*. Typhimurium, simultaneously.

### Protective efficacy of live attenuated *S*. *enterica* vaccines against wild-type *S*. Typhimurium and *S*. Enteritidis

**Fig 8:**
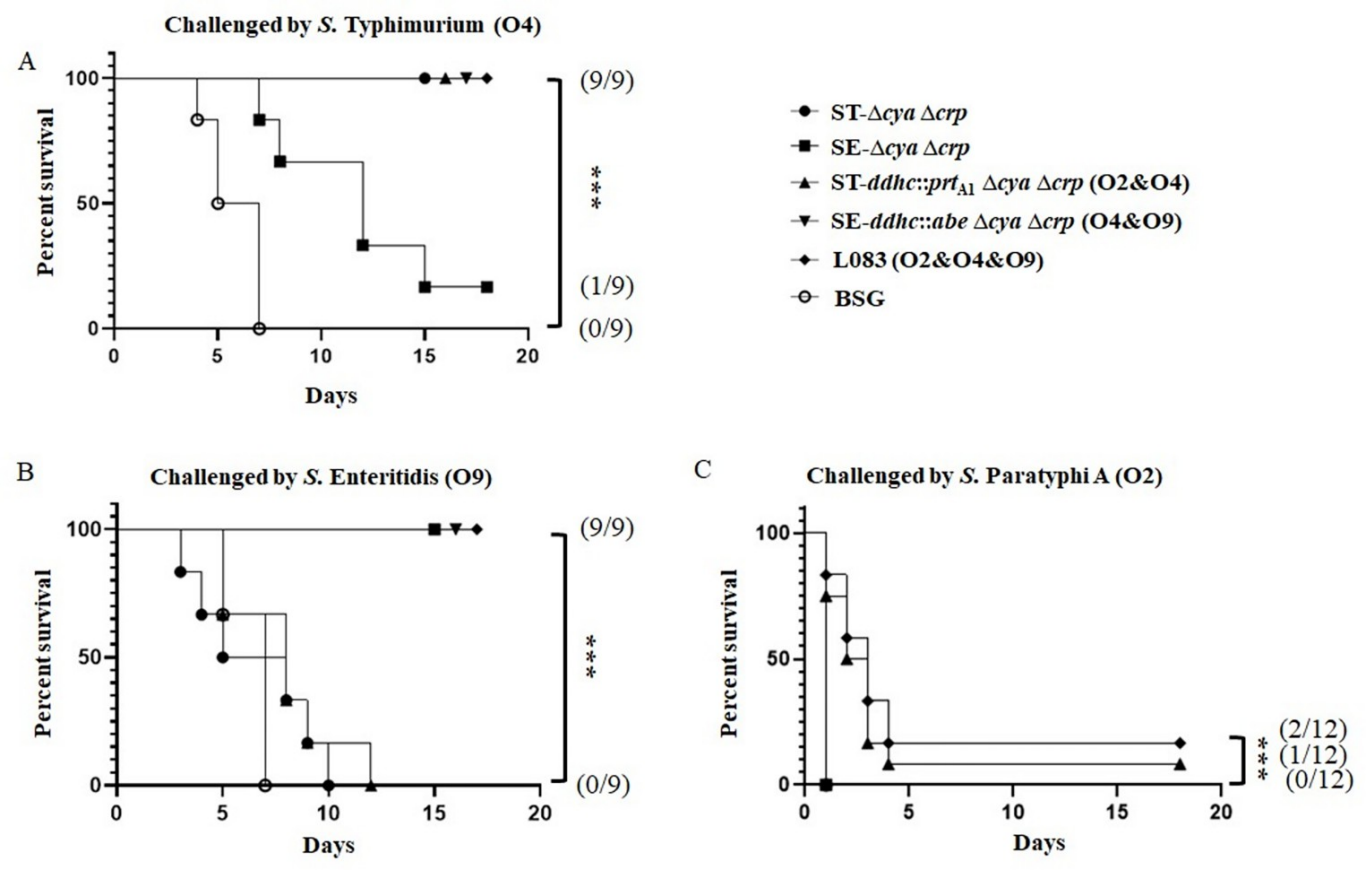
Survival curves after a challenge by wild-type virulent *Salmonella*. Five weeks after primary immunization, nine to twelve mice from each group were challenged orally or intraperitoneally with approximately 100 times the LD_50_ of the wild-type virulent *S*. Typhimurium (A), *S*. Enteritidis (B) and *S*. Paratyphi A (C). Curve comparisons were calculated using GraphPad Prism by comparing two groups by the log-rank (Mantel-Cox) test for all the marked groups versus the PBS group. ***, P<0.001. The numbers in brackets represented the number of mice alive at the end of the experiment/number of mice challenged.

The immune protection against wild-type *S*. Paratyphi A, *S*. Typhimurium and *S*. Enteritidis were evaluated in the murine model (Fig 8). Unfortunately, most of the vaccinated mice succumbed to the 100 times LD_50_ challenge from *S*. Paratyphi A. Only three mice, one out of twelve in L008 (O2&O4) and two out of twelve in L083 (O2&O4&O9) vaccinated group, survived in the end. However, mice vaccinated by L008 (O2&O4) and L083 (O2&O4&O9) could generally survive as long as 4 days, while the other vaccinated groups could barely survive a single day, showing a positive correlation of cross-protection. Meanwhile, mice vaccinated by ST-Δ*crp* Δ*cya* (O4) or SE-Δ*crp* Δ*cya* (O9) could survive a 100 times LD_50_ challenge from *S*. Typhimurium or *S*. Enteritidis, respectively, indicating a high level of homologous protection. However, these mice succumbed to the heterogenous challenge. Intriguingly, mice vaccinated by L009 (O4&O9) could independently survive a 100 times LD_50_ challenge from *S*. Typhimurium and *S*. Enteritidis, indicating a good cross-protection against heterogenous challenge. Similar results were also observed in mice vaccinated by L083 (O2&O4&O9). These mice could independently survive a 100 times LD_50_ challenge from *S*. Typhimurium and *S*. Enteritidis. Taken together, our vaccine confidants possessed good abilities to elicit cross-immune responses and cross-protections.

## Discussion

Traditionally, *Salmonella* vaccine developments followed the strategies of targeting one particular serovar or serotype, leaving the cross-protection largely unexplored. However, recently, a bivalent outer membrane vesicle (GMMAs) approach targeting S. Enteritidis and S. Typhimurium [19] and a trivalent glycoconjugate approach targeting S. Enteritidis, S. Typhimurium, and S. Typhi [21] are showing the light of multi-valent strategies. In this study, we explored another way of improving the cross-protections against *S*. Paratyphi A, *S*. Typhimurium and *S*. Enteritidis by O-antigen O-epitopes rational design.

The antisera of patients who recovered from paratyphoid fever or iNTS diseases mainly target the O-antigen polysaccharides of *S*. enterica serovars, indicating the importance of anti-OAg antibodies in patients’ adaptive immune responses. The O-serotype specificity of *S*. Paratyphi A, *S*. Typhimurium and *S*. Enteritidis is determined by the 3,6-dideoxyhexosyl side-branch residues of the trisaccharide backbone (Figs 1 and S1) [22]. Therefore, our strategy was to exhibit double or triple immunodominant O-serotypes in one *S*. *enterica* serovar. In order to achieve that goal, we first explored the possibility of co-expression of two O-epitopes. As the results had shown, either inserting the *abe* gene in front of the *prt*_A1_ gene in *S*. Paratyphi A (SA-*ddhc*::*abe*) or the *prt*_A1_ gene in front of the *abe* gene in *S*. Typhimurium (ST-*ddhc*::*prt*_A1_) (S2 Fig), we all get an obviously bacterial agglutination with O2 and O4 antisera (Table 2). Consistently, positive bands were observed in the western blot assays when using O2 and O4 antisera as the primary antibody (Fig 2A). These data all indicated that O2 and O4 could be simultaneously expressed in either *S*. Paratyphi A or *S*. Typhimurium background. Similarly, we inserted the *abe* gene in front of the *prt*_D1_ gene in *S*. Enteritidis (SE-*ddhc*::*abe*), and the *prt*_D1_ gene in front of the *abe* gene in *S*. Typhimurium (ST-*ddhc*::*prt*_*D1*_*-tyv*_*D1*_) to testify the possibility of O4 and O9 O-serotypes exhibition (S2 Fig). We observed a clear bacterial agglutination with O4 and O9 antisera in both SE-*ddhc*::*abe* and ST-*ddhc*::*prt*_*D1*_*-tyv*_*D1*_ (Table 2). However, the western blot assay did not show anti-O9 positive bands in SE-*ddhc*::*abe* sample or anti-O4 positive bands in ST-*ddhc*::*prt*_*D1*_*-tyv*_*D1*_ sample (Fig 2B). The possible explanation for this might be the unbalanced expression of O4 and O9 O-epitopes, as this phenomenon has already existed naturally in wild-type *S*. Enteritidis. In wild-type *S*. Enteritidis or *S*. Typhi, the CDP-Par and CDP-Tyv synthesize at a similar level. However, the immunodominant O-serotype of *S*. Enteritidis or *S*. Typhi is exclusively O9, with even undetectable bacterial agglutination to O2 antiserum. On the other hand, *S*. Paratyphi A exhibiting O2 O-serotype is due to the frameshift mutation in tyv gene [47], interrupting the synthesis of CDP-Tyv from CDP-Par, leaving the *S*. Paratyphi A with no choice but utilizing the CDP-Par as its substrate for side-branch sugar attachment. Lastly, we failed to express O2, O4 and O9 O-serotypes in either *S*. Paratyphi A, *S*. Typhimurium or *S*. Enteritidis strain by chromosomally genetic modification.

It seems that *S*. *enterica* serovars have a different preference for the CDP-Abe, CDP-Par and CDP-Tyv substrates when they are synthesized at a similarly level. At least, it is evident that *S*. *enterica* prefers CDP-Tyv rather than CDP-Par [28]. Therefore, we hypothesized that achieving an O2, O4 and O9 O-serotype phenotype might be possible by increasing the synthesis of CDP-Par. So, we built a dual-plasmid expression system in a *S*. Typhimurium mutant, i.e., ST-Δ*alr* Δ*dadB* Δ*recF* Δ*asd* (pSC101-*asd* or p15a-*dadB*). As the WbaV glycosyltransferase and Wzx flippase might have influenced synthesizing and transferring of different side-branch O-units, we amplified the whole *prt*_*A1*_*-tyv*_*A1*_*-wbaV*_*A1*_*-wzx*_*A1*_ genes from *S*. Paratyphi A and inserted them into pSC101-*asd* vector, resulting in pSC101-*asd-*O2 in brief (S2 Fig). At the same time, we amplified the whole *prt*_*D1*_*-tyv*_*D1*_*-wbaV*_*D1*_*-wzx*_*D1*_ genes from *S*. Enteritidis and inserted them into p15a-*dadB* vector, resulting in p15a-*dadB-*O9 in brief. Predictably, ST-Δ*alr* Δ*dadB* Δ*recF* Δ*asd* harboring both pSC101-*asd-*O2 and p15a-*dadB-*O9 would increase CDP-Par synthesis, as there are at least two copies of *prt* gene transcribing but with only one normal *tyv* gene. Meanwhile, the synthesis of CDP-Abe was unaffected. The bacterial agglutination assays demonstrated that ST-Δ*alr* Δ*dadB* Δ*recF* Δ*asd* (pSC101-*asd-*O2, p15a-*dadB-*O9) could agglutinate with O2, O4 and O9 antiserum individually, indicating that we had successfully achieved a triple immunodominant O-serotypes phenotype in *S*. Typhimurium background (Table 2). However, we did not receive a consistent result from western blot assays, which showed a predominantly anti-O9 positive band (Fig 2C). This inconsistency between the bacterial agglutination and western blot assay might indicate the amount or distribution of O2, O4 and O9 O-epitopes in ST-Δ*alr* Δ*dadB* Δ*recF* Δ*asd* (pSC101-*asd*-O2, p15a-*dadB-*O9) is most likely unequal or unbalanced. We could not explain clearly how these O-epitopes are arranged in the outer membrane, either homogeneously or heterogeneously attached to each of the O-antigen polysaccharides (S7 Fig), or merely mixed irregularity. It is also possible that new irrelevant epitopes are created and, in this case, a portion of the antibody response may not be functional.

Liu etc. had shown that the glycosyltransferase WbaV of *S*. *enterica* is in fact more effective in attaching the Tyv to the O-antigen common trisaccharide backbone than Par, and this inefficiency of low Par utilization could be primarily improved by overexpressing the wbaV gene [28]. Their findings were reconfirmed in this study. Meanwhile, Hong etc. had also shown that the Wzx flippases have a strong preference for their cognate substrate [29,30]. Furthermore, they demonstrated that the absence of the side-branch resides would dramatically decrease the efficiency of Wzx flippases [29]. However, no direct evidence had been put forward to illustrate the influence of different side-branch resides upon Wzx translocation efficiency. Our previous report demonstrated that the *S*. Typhimurium could tolerate well Tyv side-branch O-units but reject Par side-branch O-units [32], indicating that nonnative substrate does influence Wzx translocation efficiency. However, in this study, we further developed these theories by showing that *S*. Typhimurium did not exhibit severe rejection against CDP-Par if accompanied by CDP-Abe. Moreover, *S*. Typhimurium could utilize CDP-Par as its side-branch building block even in the presence of CDP-Tyv.

After successfully constructing a series of double or triple immunodominant O-serotype mutant strains, we would like to know their phenotype characterizations. The phage P22 tailspike protein recognizes and hydrolyzes the repetitive O-antigen polysaccharides of *S*. *enterica* serogroup A, B and D at the Rha-Gal (1→3)-glycosidic linkages during infections [48]. So, we first performed a P22 phage transduction assay to check the O-antigen integrality of our mutant strains. The transduction numbers of our constructed mutants were similar to their wild-type parent strains (Table 3), which indicated that our insertion mutations did not change the essential common trisaccharide backbone of O-antigen polysaccharides. Next, the LPS profile of each mutant strain was examined. It is obvious that ST-*ddhc*::*prt*_A1_ and SE-*ddhc*::*abe* mutants could exhibit a full length of LPS (Fig 3A), while ST-Δ*alr* Δ*dadB* Δ*recF* Δ*asd* (pSC101-*asd*-O2, p15a-*dadB*) or ST-Δ*alr* Δ*dadB* Δ*recF* Δ*asd* (pSC101-*asd*, p15a-*dadB-*O9) showed a decreased synthesis of LPS length (Fig 3B). Surprisingly, ST-Δ*alr* Δ*dadB* Δ*recF* Δ*asd* (pSC101-*asd-*O2, p15a-*dadB*-O9) exhibited a full length of LPS synthesis, indicating a higher level of CDP-Par synthesis might be the critical factor in achieving a triple O-serotype phenotype in a *S*. *enterica* strain. However, this assumption is not strictly demonstrated in this study. Meanwhile, we also conducted other phenotype evaluation assays (Table 3), such as the DOC and polymyxin B MICs, to roughly mimic the intestinal environment of the natural oral infection route and swimming abilities, an indicator of bacteria surface “wettability” [49]. After all these tests, we selected ST-*ddhc*::*prt*_A1_, SE-*ddhc*::*abe* and ST-Δ*alr* Δ*dadB* Δ*recF* Δ*asd* (pSC101-*asd*-O2, p15a-*dadB-*O9) for further cross-immunogenicity evaluation. To guarantee their safety in the murine model, the global regulator *cya* and *crp* were deleted in these mutant strains [50], resulting in a series of potential vaccine candidates. The vaccine candidates showed good colonization in Peyer’s patches, livers, and spleens (Fig 5).

We are primarily interested in whether or not our live attenuated vaccine candidates could elicit effective cross-immune responses. To evaluate that, we applied ELISA assays to detect the antibodies raised against the O-antigen polysaccharides of wild-type *S*. Paratyphi A, *S*. Typhimurium and *S*. Enteritidis (Fig 6). Consistent with the bacterial agglutination assays, L008 (O2&O4) could induce a significantly higher anti-O2 antibody response than ST-Δ*crp* Δ*cya* (O4). Meanwhile, L009 (O4&O9) could induce a significantly higher anti-O4 antibody response than SE-Δ*crp* Δ*cya* (O9). Most importantly, L083 (O2&O4&O9) could simultaneously induce significantly higher anti-O2, anti-O4 and anti-O9 antibody responses in mice when compared to the negative control, SE-Δ*crp* Δ*cya* (O9) and ST-Δ*crp* Δ*cya* (O4), respectively. These data showed that our vaccine candidates could elicit effective cross-immune responses. Although a high level of anti-*S*. Typhimurium, anti-*S*. Enteritidis and anti-*S*. Paratyphi A LPS serum antibodies were obtained, we would like to know whether or not these raised antibodies are indeed functional. The *in vitro* bactericidal data indicated that serum complement-mediated *S*. Paratyphi A, *S*. Typhimurium and *S*. Enteritidis killing depended upon anti-O2, anti-O4 and anti-O9 antibodies (Fig 7). Moreover, we challenged all vaccinated mice with wild-type virulent *S*. Paratyphi A, *S*. Typhimurium or *S*. Enteritidis strain (Fig 8). Compared with the negative control, ST-Δ*crp* Δ*cya* (O4) and SE-Δ*crp* Δ*cya* (O9) vaccinated mice could receive good homologous protection but failed to heterologous challenge. However, mice vaccinated by double or triple O-epitopes vaccine strains received a significantly higher protection rate even when challenged with heterologous wild-type virulent strains, showing adequate cross-immunity protection.

However, there are some limitations to this study. The *S*. Typhimurium and *S*. Enteritidis used in SBA assay are all animal isolates, which could not simply be equal to the human blood isolates. Other reports have shown that anti-LPS antibodies stimulate low complement-dependent killing against S. Enteritidis human blood isolates [51]. Furthermore, S. Typhimurium ST313, which dominates in sub-Saharan Africa, is highly serum resistant and expresses distinct transcriptional patterns that may aid in escaping this killing mechanism [52]. It is a good future research direction to illustrate how they escape this complement-dependent killing mechanism.

In summary, we expressed double or triple immunodominant O-epitopes in a *S*. Typhimurium or *S*. Enteritidis background strain and proved they are effective in inducing cross-immunity and cross-protection. Significantly, the strategies we present in this study are not limited to *S*. *enterica* O-antigen polysaccharides but have applicability for generating cross-protection for many other important human and animal pathogens. Notable examples include *Shigella flexneri* and many pathogenic *Escherichia coli*.

## Supporting information

S1 Fig The O-antigen gene cluster comparisons of *S*. Paratyphi A, *S*. Enteritidis and *S*. Typhimurium. The O-antigen gene clusters are within the *galF* and *gnd* genes of *S*. Paratyphi A, *S*. Enteritidis and *S*. Typhimurium genome, which could be accessed through the genebank accession numbers NZ_CP019185.1, CP007361.1 and CP002614.1, respectively. The O-antigen gene clusters of groups A1 and D1 are highly homologous. The main differences between group B1 and group A1, D1 are the regions responsible for synthesizing the side-branch sugars. Note that the tyvA1 has a loss-of-function mutation due to ORF frameshift. Diagrams are drawn to scale.
(TIF)

S2 Fig Schematic representation of insertion mutations and plasmid constructions. The *abe* gene from *S*. Typhimurium was inserted between the *ddhc* and *prt*_A1_ gene of *S*. Paratyphi A. (B) The *prt*_A1_ gene was inserted between the *ddhc* and *abe* gene of *S*. Typhimurium. (C) The *abe* gene was inserted between the *ddhc* and *prt*_D1_ gene of *S*. Enteritidis. (D) The *prt*_D1_-*tyv*_D1_ genes were inserted between the *ddhc* and *abe* gene of *S*. Typhimurium. (E) The *prt*_A1_-*tyv*_A1_-*wbaV*_A1_-*wzx*_A1_ genes from *S*. Paratyphi A were cloned into pSC101-*asd*, resulting in pSC101-*asd*-O2. (F) The *prt*_D1_-*tyv*_D1_-*wbaV*_D1_-*wzx*_D1_ genes from *S*. Enteritidis were cloned into p15a-*dadB*, resulting in p15a-*dadB*-O9. Primer pairs used for each DNA fragment amplification were labeled accordingly.
(TIF)

S3 Fig The bacterial agglutination assays. The agglutination assays were performed on glass slides and the used anti-Par O2, anti-Abe O4 and anti-Tyv O9 antiserum were indicated above. Positive or negative agglutination could be observed directly by the naked eye. Images were taken at 10 × 10 magnification.
(TIF)

S4 Fig Serum IgG2a and IgG1 responses. Serum IgG2a and IgG1 responses against the LPS of *S*. Paratyphi A (A), *S*. Enteritidis (B) and *S*. Typhimurium (C) were determined by ELISA. A significantly higher level of IgG2a specific to the *S*. Paratypi A LPS compared to IgG1 was observed in L008 (O2&O4) and L083 (O2&O4&O9) (***, P<0.001). A significantly higher level of IgG2a specific to the *S*. Enteritis LPS compared to IgG1 was observed in SE-Δ*crp* Δ*cya* (***, P<0.001). A significantly higher level of IgG2a specific to the *S*. Typhimurium LPS compared to IgG1 was observed in ST-Δ*crp* Δ*cya*, L008 (O2&O4), L009 (O4&O9) and L083 (O2&O4&O9) (***, P<0.001). The antibody concentrations were calculated using a standard curve. All of the measured sample concentrations were within the standard curve range. The error bars represent the standard deviation of the means. These data are representative of at least two independent experiments.
(TIF)

S5 Fig Serum IgM responses. (A) Anti-*S*. Paratyphi A LPS serum IgM levels. Responses that differed from the results in the ST-Δ*cya* Δ*crp* group are noted by asterisks (***, P<0.001). (B) The anti-*S*. Enteritidis LPS serum IgM levels. Responses that differed from the results in the ST-Δ*cya* Δ*crp* group are noted by asterisks (***, P<0.001). (C) Anti-*S*. Typhimurium LPS serum IgM levels. Responses that differed from the results in the SE-Δ*cya* Δ*crp* group are noted by asterisks (***, P<0.001). Antibody concentrations were calculated using a standard curve and all the measured sample concentrations were within the standard curve range. The error bars represent the standard deviation of the means. These data are representative of at least two independent experiments.
(TIF)

S6 Fig IgA antibody responses in mice vaginal secretions. (A) Anti-*S*. Paratyphi A LPS serum IgA levels. Responses that differed from the results in the ST-Δ*cya* Δ*crp* group are noted by asterisks (*, P<0.05; **, P<0.01). (B) The anti-*S*. Enteritidis LPS serum IgA levels. Responses that differed from the results in the ST-Δ*cya* Δ*crp* group are noted by asterisks (***, P<0.001). (C) Anti-*S*. Typhimurium LPS serum IgA levels. Responses that differed from the results in the SE-Δ*cya* Δ*crp* group are noted by asterisks (**, P<0.01). Antibody concentrations were calculated using a standard curve and all the measured sample concentrations were within the standard curve range. The error bars represent the standard deviation of the means calculated by GraphPad Prism software. These data are representative of at least two independent experiments.
(TIF)

S7 Fig A schematic diagram of *S*. Typhimurium outer membrane O-antigen epitopes arrangement. (A) The O2, O4 and O9 O-epitopes are homogeneously attached to each one of the O-antigen polysaccharides. (B) The O2, O4 and O9 O-epitopes are heterogeneously attached to each one of the O-antigen polysaccharides. Note that the number of each attached O-epitope does not represent the real case.
(TIF)

S1 Text Supplementary Methods. Methods for LPS preparation and western blotting, the bacterial slide agglutination test, P22 transduction studies, motility test and minimum inhibitory concentration (MIC) test are described in supporting information supplementary methods.
(DOCX)

S1 Table Primers used in this work. (XLSX)
