# Supplementary figures and images for: Multiple immunodominant O-epitopes co-expression in live attenuated Salmonella serovars induce cross-protective immune responses against S. Paratyphi A, S. Typhimurium and S. Enteritidis
Source: PLoS Negl Trop Dis. 2022 Oct 13;16(10):e0010866. doi: 10.1371/journal.pntd.0010866 (PMC9595534; doi:10.1371/journal.pntd.0010866)

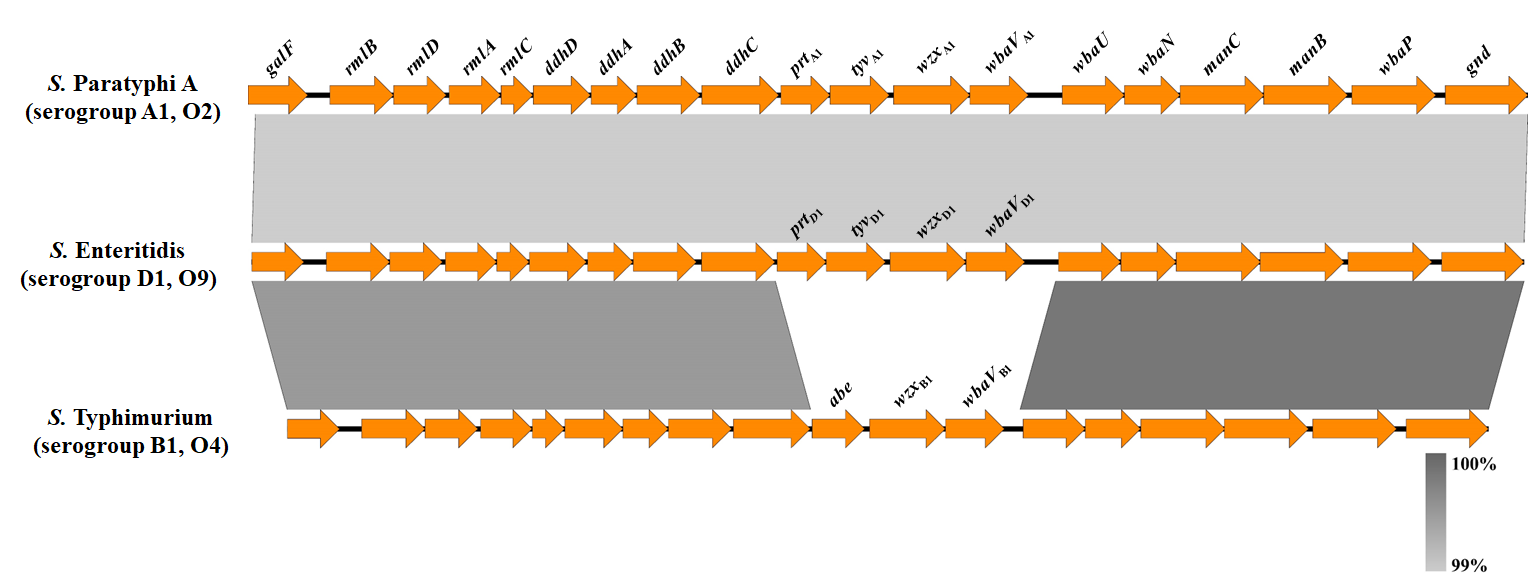

Supplement: S1 Fig — The O-antigen gene clusters are within the galF and gnd genes of S. Paratyphi A, S. Enteritidis and S. Typhimurium genome, which could be accessed through the genebank accession numbers NZ_CP019185.1, CP007361.1 and CP002614.1, respectively. The O-antigen gene clusters of groups A1 and D1 are highly homologous. The main differences between group B1 and group A1, D1 are the regions responsible for synthesizing the side-branch sugars. Note that the tyvA1 has a loss-of-function mutation due to ORF frameshift. Diagrams are drawn to scale. (TIF) [file pntd.0010866.s001.tif]

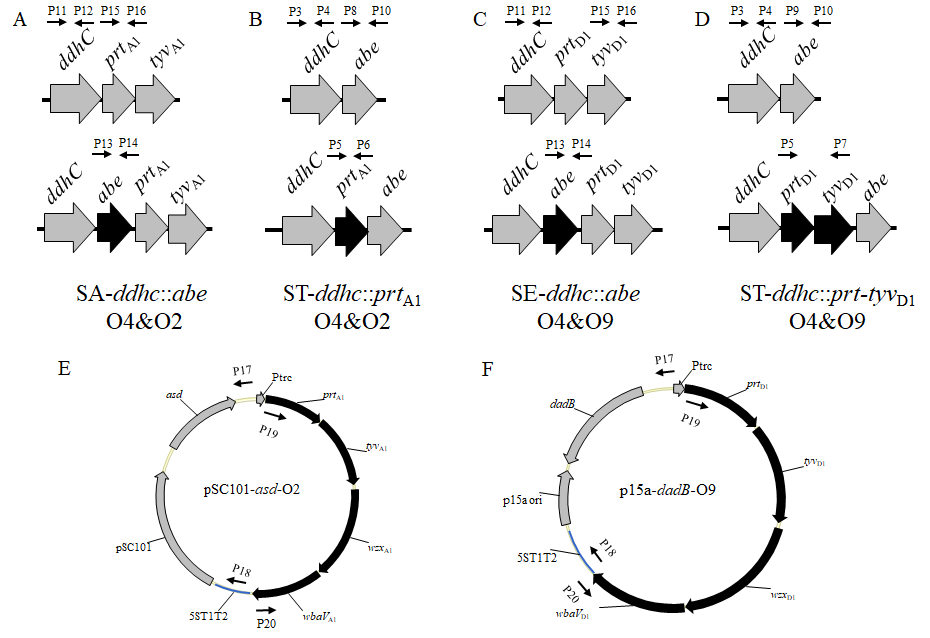

Supplement: S2 Fig — The abe gene from S. Typhimurium was inserted between the ddhc and prtA1 gene of S. Paratyphi A. (B) The prtA1 gene was inserted between the ddhc and abe gene of S. Typhimurium. (C) The abe gene was inserted between the ddhc and prtD1 gene of S. Enteritidis. (D) The prtD1-tyvD1 genes were inserted between the ddhc and abe gene of S. Typhimurium. (E) The prtA1-tyvA1-wbaVA1-wzxA1 genes from S. Paratyphi A were cloned into pSC101-asd, resulting in pSC101-asd-O2. (F) The prtD1-tyvD1-wbaVD1-wzxD1 genes from S. Enteritidis were cloned into p15a-dadB, resulting in p15a-dadB-O9. Primer pairs used for each DNA fragment amplification were labeled accordingly. (TIF) [file pntd.0010866.s002.tif]

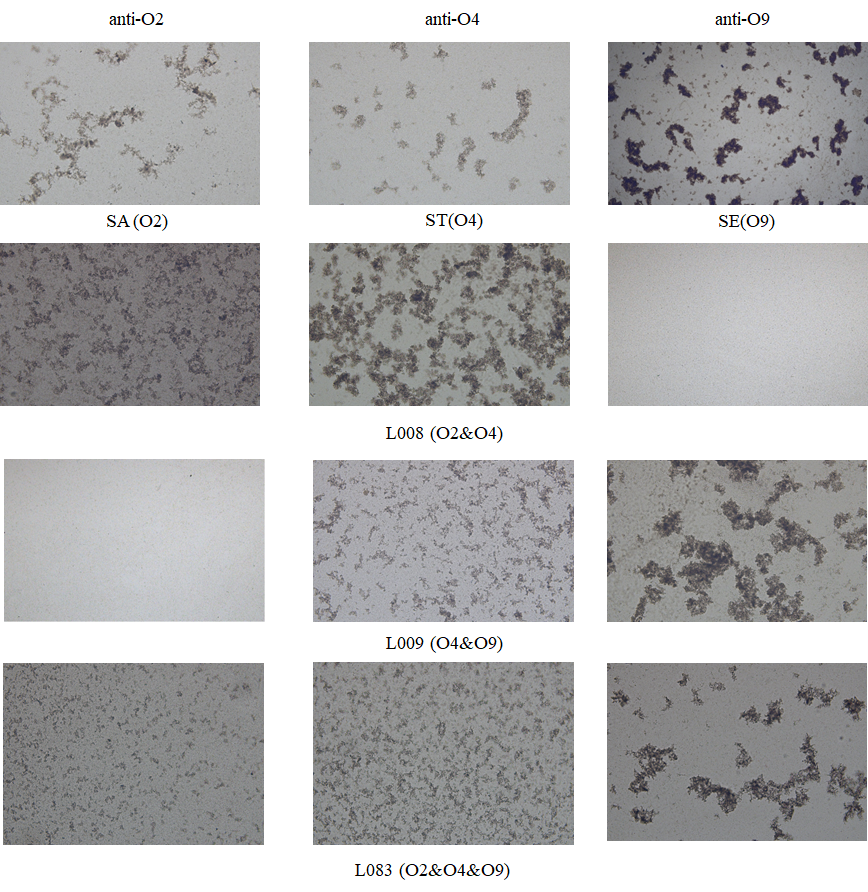

Supplement: S3 Fig — The agglutination assays were performed on glass slides and the used anti-Par O2, anti-Abe O4 and anti-Tyv O9 antiserum were indicated above. Positive or negative agglutination could be observed directly by the naked eye. Images were taken at 10 × 10 magnification. (TIF) [file pntd.0010866.s003.tif]

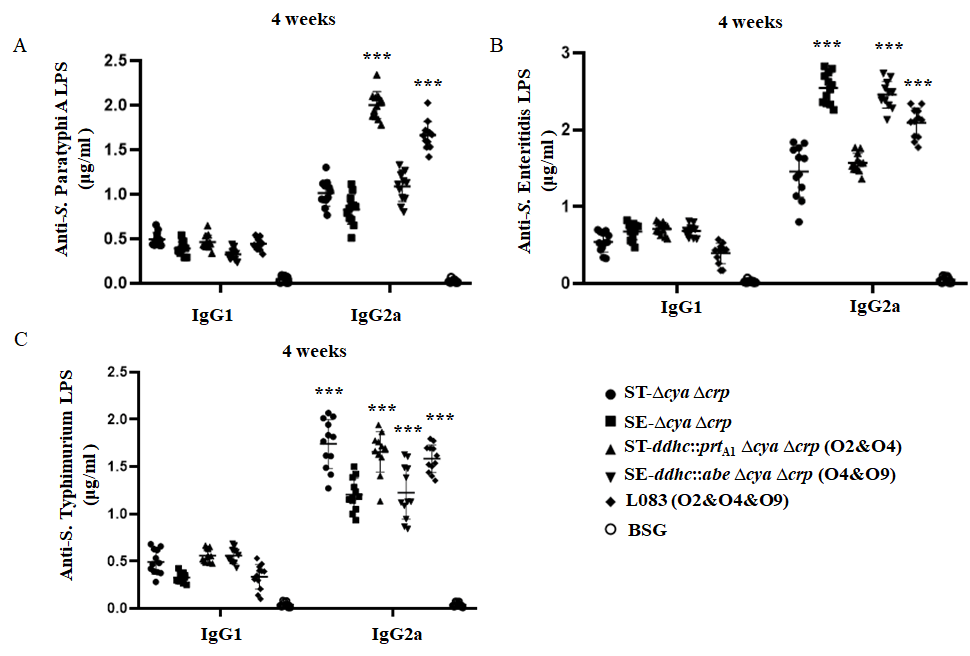

Supplement: S4 Fig — Serum IgG2a and IgG1 responses against the LPS of S. Paratyphi A (A), S. Enteritidis (B) and S. Typhimurium (C) were determined by ELISA. A significantly higher level of IgG2a specific to the S. Paratypi A LPS compared to IgG1 was observed in L008 (O2&O4) and L083 (O2&O4&O9) (***, P<0.001). A significantly higher level of IgG2a specific to the S. Enteritis LPS compared to IgG1 was observed in SE-Δcrp Δcya (***, P<0.001). A significantly higher level of IgG2a specific to the S. Typhimurium LPS compared to IgG1 was observed in ST-Δcrp Δcya, L008 (O2&O4), L009 (O4&O9) and L083 (O2&O4&O9) (***, P<0.001). The antibody concentrations were calculated using a standard curve. All of the measured sample concentrations were within the standard curve range. The error bars represent the standard deviation of the means. These data are representative of at least two independent experiments. (TIF) [file pntd.0010866.s004.tif]

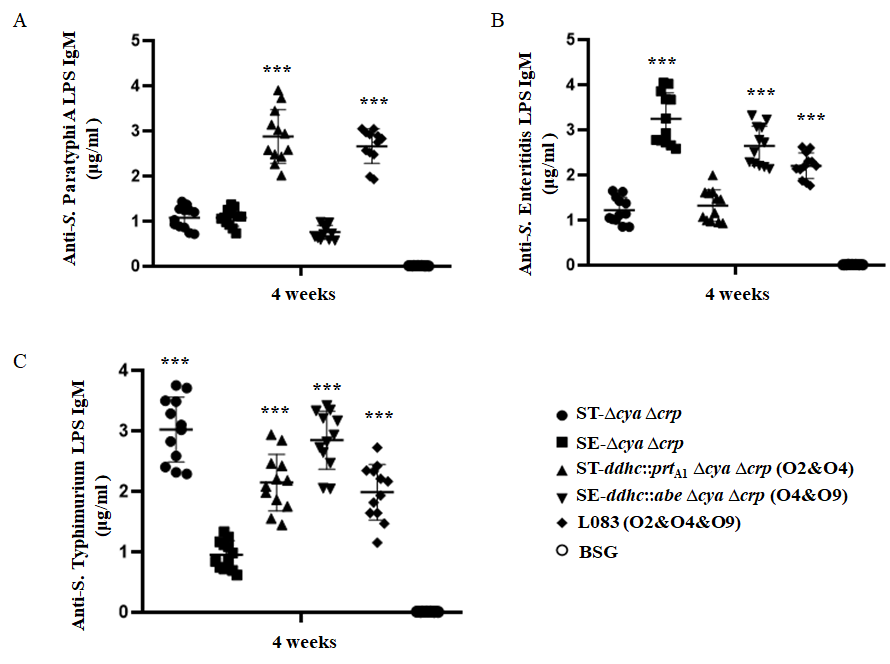

Supplement: S5 Fig — (A) Anti-S. Paratyphi A LPS serum IgM levels. Responses that differed from the results in the ST-Δcya Δcrp group are noted by asterisks (***, P<0.001). (B) The anti-S. Enteritidis LPS serum IgM levels. Responses that differed from the results in the ST-Δcya Δcrp group are noted by asterisks (***, P<0.001). (C) Anti-S. Typhimurium LPS serum IgM levels. Responses that differed from the results in the SE-Δcya Δcrp group are noted by asterisks (***, P<0.001). Antibody concentrations were calculated using a standard curve and all the measured sample concentrations were within the standard curve range. The error bars represent the standard deviation of the means. These data are representative of at least two independent experiments. (TIF) [file pntd.0010866.s005.tif]

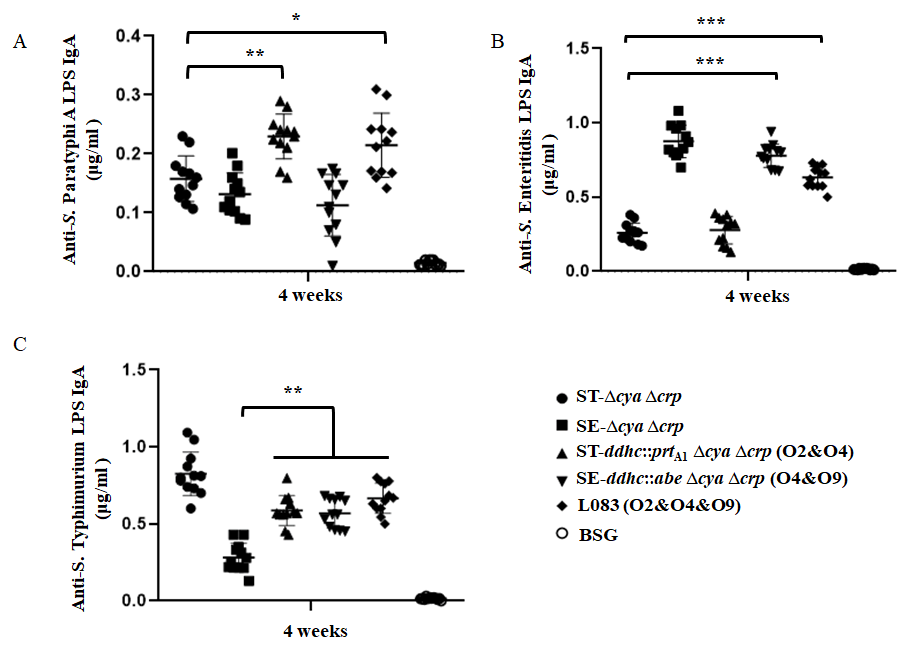

Supplement: S6 Fig — (A) Anti-S. Paratyphi A LPS serum IgA levels. Responses that differed from the results in the ST-Δcya Δcrp group are noted by asterisks (*, P<0.05; **, P<0.01). (B) The anti-S. Enteritidis LPS serum IgA levels. Responses that differed from the results in the ST-Δcya Δcrp group are noted by asterisks (***, P<0.001). (C) Anti-S. Typhimurium LPS serum IgA levels. Responses that differed from the results in the SE-Δcya Δcrp group are noted by asterisks (**, P<0.01). Antibody concentrations were calculated using a standard curve and all the measured sample concentrations were within the standard curve range. The error bars represent the standard deviation of the means calculated by GraphPad Prism software. These data are representative of at least two independent experiments. (TIF) [file pntd.0010866.s006.tif]

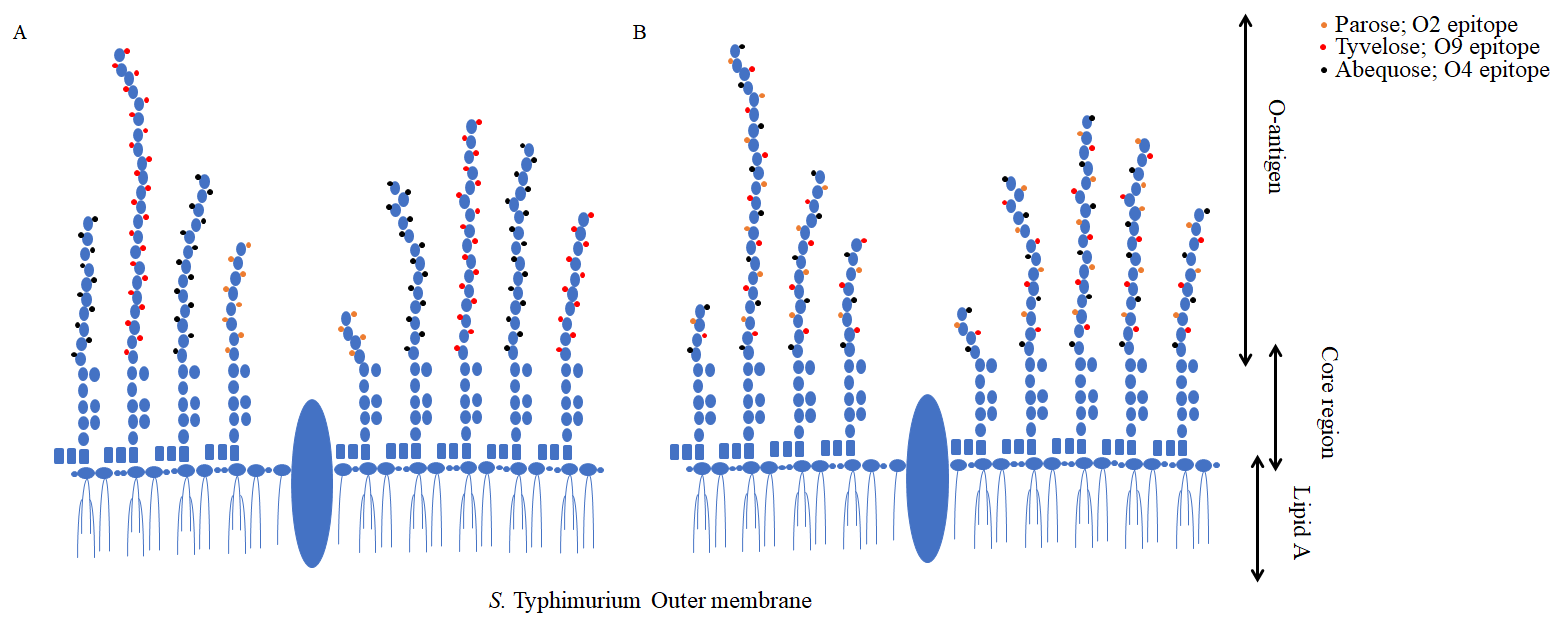

Supplement: S7 Fig — (A) The O2, O4 and O9 O-epitopes are homogeneously attached to each one of the O-antigen polysaccharides. (B) The O2, O4 and O9 O-epitopes are heterogeneously attached to each one of the O-antigen polysaccharides. Note that the number of each attached O-epitope does not represent the real case. (TIF) [file pntd.0010866.s007.tif]
