# Supplementary material for: Multiple immunodominant O-epitopes co-expression in live attenuated Salmonella serovars induce cross-protective immune responses against S. Paratyphi A, S. Typhimurium and S. Enteritidis
Source: PLoS Negl Trop Dis. 2022 Oct 13;16(10):e0010866. doi: 10.1371/journal.pntd.0010866 (PMC9595534; doi:10.1371/journal.pntd.0010866)
Supplement: S1 Text — Methods for LPS preparation and western blotting, the bacterial slide agglutination test, P22 transduction studies, motility test and minimum inhibitory concentration (MIC) test are described in supporting information supplementary methods. (DOCX) [file pntd.0010866.s008.docx]

**Supplementary Methods**

**LPS preparation and western blotting**

Firstly, *Salmonella* was grown at 37 ℃ in Luria-Bertani (LB, Oxoid Ltd.) broth at 220 rpm until they reached the OD_600_ of 0.8. Bacteria suspension (around 2 x 10^8^ CFU/mL in 2 mL PBS) were used for the LPS extraction. LPS samples were prepared, separated and visualized using the method provided by Hitchcock and Brown [1]. The same extracted LPS sample (8 μl) was used for both the LPS silver staining and LPS Western blot. As for the LPS western blotting, the samples were transferred to PVDF membranes using a Trans-Blot SD semidry transfer system (Bio-Rad). The O-antigen signal-factor rabbit antisera (BD Biosciences) (1:500 dilution) was used as the primary antibody and then followed by the secondary anti-rabbit alkaline phosphatase-conjugated antibody (Sigma) at a 1:1000 dilution. The primary antibody was incubated overnight at 4 ℃, while the secondary antibody was incubated 2 h at room temperature. Patterns were visualized using nitroblue tetrazolium-5-bromo-4-chloro-3-indolylphosphate (Sigma).

**The bacterial slide agglutination test**

The slide agglutination test is done on a glass slide and read with the naked eye in front of a light source against a black background. Add a small drop of antiserum (BD Biosciences) on a glass slide and mix it with *Salmonella* culture. Tilt the slide. A positive reaction is seen as visible agglutination, whereas a negative reaction is seen as homogeneous milky turbidity. Images were taken at 10 × 10 magnification.

**P22 transduction studies**

Evaluation of phage P22 transduction efficacy was performed as described previously[2]. Briefly, P22HT *int* was propagated in *S*. Typhimurium S100 carrying the chromosomal-integrated chloramphenicol resistance suicide vector pSS241. The recipient strains being tested were grown to an OD_600_ of 0.6 (~5 × 10^6^ CFU/ml), and 10 µl of the diluted phage (1 × 10^8^ PFU) was mixed with 1 ml of bacteria and incubated at 37°C for 30 min. After incubation, the mixture was centrifuged and resuspended in 1 ml of PBS. A 100-µl aliquot was spread on an LB agar plate containing 25 µg/ml chloramphenicol. After the plates were incubated at 37°C overnight, colonies were counted. This experiment was repeated three times.

**Motility test**

Motility tests were performed on 0.3% soft agar overlays. Briefly, LB soft plates were dried at room temperature for approximately 2 h prior to performing the assays. 6 µl of freshly grown bacteria (~5 × 10^6^ CFU) was pipetted onto the center of the plates, which were then incubated at 37°C for 6 h prior to the colony diameters (in millimeters) being measured. This experiment was repeated three times.

**Minimum inhibitory concentration (MIC) test**

The MICs of deoxycholate (DOC) and polymyxin B were determined using 96-well microtiter plates. Two-fold serial dilutions of DOC (0.312–80 mg/ml) and polymyxin B (0.072–9.2 µg/ml) were made along the plates. Bacteria were grown to an OD_600_ of 0.6 and diluted to ~5.0×10^4^ CFU/ml in LB broth. Then, 100 µl of the diluted bacteria suspension was added to each well, followed by overnight incubation at 37°C. The optical density of each well was determined using an iMark^TM^ microplate reader (Bio-Rad). The threshold of inhibition was 0.1 at OD_600_. This assay was repeated three times.

**Reference**

1. Hitchcock PJ, Brown TM. Morphological heterogeneity among Salmonella lipopolysaccharide chemotypes in silver-stained polyacrylamide gels. J Bacteriol. 1983;154(1):269-77.

2. Li P, Liu Q, Luo H, Liang K, Yi J, Luo Y, et al. O-Serotype Conversion in Salmonella Typhimurium Induces Protective Immune Responses against Invasive Non-Typhoidal Salmonella Infections. Front Immunol. 2017;8:1647. Epub 2017/12/20. doi: 10.3389/fimmu.2017.01647. PubMed PMID: 29255460; PubMed Central PMCID: PMCPMC5722840.
